# Increased Firing Irregularity as an Emergent Property of Neural-State Transition in Monkey Prefrontal Cortex

**DOI:** 10.1371/journal.pone.0080906

**Published:** 2013-12-04

**Authors:** Kazuhiro Sakamoto, Yuichi Katori, Naohiro Saito, Shun Yoshida, Kazuyuki Aihara, Hajime Mushiake

**Affiliations:** 1 Research Institute of Electrical Communication, Tohoku University, Sendai, Japan; 2 Institute of Industrial Science, University of Tokyo, Tokyo, Japan; 3 Funding Program for World-Leading Innovative Research and Development on Science and Technology, Aihara Innovative Mathematical Modelling Project, Japan Science and Technology Agency, Tokyo, Japan; 4 Department of Physiology, Tohoku University School of Medicine, Sendai, Japan; 5 The Core Research for Evolutional Science and Technology Program, Japan Science and Technology Agency, Tokyo, Japan; SUNY Downstate MC, United States of America

## Abstract

Flexible behaviors are organized by complex neural networks in the prefrontal cortex. Recent studies have suggested that such networks exhibit multiple dynamical states, and can switch rapidly from one state to another. In many complex systems such as the brain, the early-warning signals that may predict whether a critical threshold for state transitions is approaching are extremely difficult to detect. We hypothesized that increases in firing irregularity are a crucial measure for predicting state transitions in the underlying neuronal circuits of the prefrontal cortex. We used both experimental and theoretical approaches to test this hypothesis. Experimentally, we analyzed activities of neurons in the prefrontal cortex while monkeys performed a maze task that required them to perform actions to reach a goal. We observed increased firing irregularity before the activity changed to encode goal-to-action information. Theoretically, we constructed theoretical generic neural networks and demonstrated that changes in neuronal gain on functional connectivity resulted in a loss of stability and an altered state of the networks, accompanied by increased firing irregularity. These results suggest that assessing the temporal pattern of neuronal fluctuations provides important clues regarding the state stability of the prefrontal network. We also introduce a novel scheme that the prefrontal cortex functions in a metastable state near the critical point of bifurcation. According to this scheme, firing irregularity in the prefrontal cortex indicates that the system is about to change its state and the flow of information in a flexible manner, which is essential for executive functions. This metastable and/or critical dynamical state of the prefrontal cortex may account for distractibility and loss of flexibility in the prefrontal cortex in major mental illnesses such as schizophrenia.

## Introduction

The prefrontal cortex plays a crucial role in flexible decision making and behavioral planning, which are essential for adapting to ever-changing environments [1], [2]. Rapid shifts in the information encoded by prefrontal neurons seem to reflect the flexible nature of the prefrontal cortex [3]–[5]. Recent studies have focused on revealing the underlying mechanisms, particularly how local prefrontal networks change their functional connectivity in a rapid and flexible manner [3], [6]–[8].

**Figure 1:**
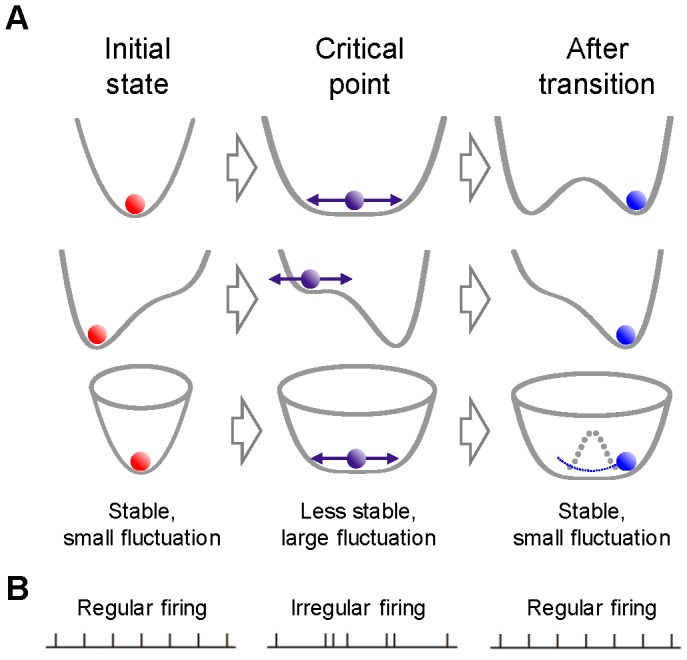
Network states and firing irregularity. (A) Schematic diagram for attractor landscapes and state transitions of dynamical systems. Each row demonstrates representative state transitions or bifurcations. From top to bottom: pitch fork, saddle-node, and Hopf bifurcations. Regardless of the type of bifurcation, dynamical systems exhibit common behavior. Far from the critical point (left), systems are resilient to perturbations, but when systems are closer to the critical point (middle), they lose resilience, become sensitive to perturbations, and are accompanied by increased variability of measurements. Following the transition (right), systems again become stable. (B) The stability of neural networks is hypothesized to be reflected in firing irregularity of constituent neurons.

From the viewpoint of dynamical-systems theory, these rapid changes in functional connectivity can be considered attractor dynamics, or state transitions [3], [7], [9]–[11]. In a wide range of complex, dynamic systems, transient increase fluctuations, referred to as critical fluctuations, are early-warning signals that can be detected prior to state transitions [12]–[16] (Fig. 1A). Specifically, dynamical systems become sensitive to perturbations and often exhibit increases in fluctuations immediately before state transitions. However, no experimental studies have attempted to determine whether prefrontal neurons exhibit increased transient fluctuations in their firing patterns before rapid shifts in the representation of neuronal information. Thus, the relationship between neuronal firing fluctuations and changes in the functional connectivity of neuronal circuits in the prefrontal cortex remains unclear.

Fluctuations in neuronal firing, measured by examining firing irregularity, could be derived from the local and/or network states of neurons. As a local factor, firing irregularity reflects the state of a single neuron receiving balanced inputs from excitatory and inhibitory neuronal inputs [17]–[19]. When excitatory and inhibitory inputs to a neuron are balanced, no net constant drift drives the membrane potential; instead, only variability in the inputs or noise modulates membrane potential [18], [20]. However, these reports focused on the synaptic or single-neuronal level. As a network factor, firing irregularity reflects the stability of the neural network, depending on functional connectivity (Fig. 1B). Dynamical neuronal networks often fall into a steady state or an attractor, and the degree of attractor stability varies depending on the gain functions of constituent neurons. When functional connectivity of the network allows a stable point attractor, networks maintain relatively regular firings, with small transient irregularity in response to perturbations. In contrast, when the network is less stable, approaching state transition or bifurcation, it becomes more susceptible to perturbations because of the instability of the network state. The network could be less stable depending on subtle changes in functional connectivity, even if each neuron receives the same balanced excitatory and inhibitory inputs. Thus, from the viewpoint of dynamical-systems theory, we hypothesize that increased firing irregularity is a crucial measure of network stability that can be used to predict state transitions in underlying neuronal circuits in the prefrontal cortex.

To test this hypothesis, we experimentally examined whether prefrontal neurons exhibit increases in firing irregularity when neural representation abruptly changes. Prefrontal neurons showed increased firing irregularity prior to switching neural encoding of behavioral goals. Next, we demonstrated theoretically that such transient increases in firing irregularity could emerge from changes in gain functions by decreasing neural network stability through state transitions or bifurcations. These results suggest that firing irregularity, neuronal gains, and attractor stability are linked in the dynamical neural networks in the prefrontal cortex that underlie the flexible and rapid adaptation to ever-changing environments. Based on these findings, we propose a new scheme that the prefrontal cortex functions in a metastable state near the critical point of bifurcation. We discuss the significance of this scheme, which may account for abnormal executive functions in major mental illnesses such as schizophrenia.

## Materials and Methods

### Subjects and Ethics

Two Japanese monkeys (*Macaca fuscata*) were used for this study. All experimental protocols were approved by the Animal Care and Use Committee, Tohoku University (Permit # 20MeA-2), and all animal protocols conformed with the National Institutes of Health guidelines for the care and use of laboratory animals and with the recommendations of the Weatherall Report. The animals were housed in adjoining individual primate cages in an air-conditioned room. Food was always available and supplementary vegetables and fruit were provided daily. Animals were provided with environmental enrichment and were permitted rich visual, olfactory and auditory interactions. To achieve adequate environmental richness, we provide toys which are easily manipulated by the animals and when they are beginning to lose interests in old toys, we introduce novel objects as toys. Throughout the study, the animals were monitored daily by the researchers and an animal research technician or veterinary technician for evidence of disease or injury and body weight was also documented daily. Animals were humanely euthanized by anesthetizing with an overdose of pentobarbital according to endpoint criteria. The endpoints are defined in our protocol as following two cases: 1) When scientific objects of the protocol are achieved by recordings neural activities from all of cortical areas of our research interest, or 2) when the animals are not able to maintain basic performance because they are ill or have physical deficits. In this case, we further consult the veterinarian every time it is necessary for appropriate treatment to keep animal health and if recovery from this deficit is not expected, we promptly decide that euthanasia is necessary as a mean to relieve pain or distress regardless of progress of the study.

### Behavioral Procedures

**Figure 2:**
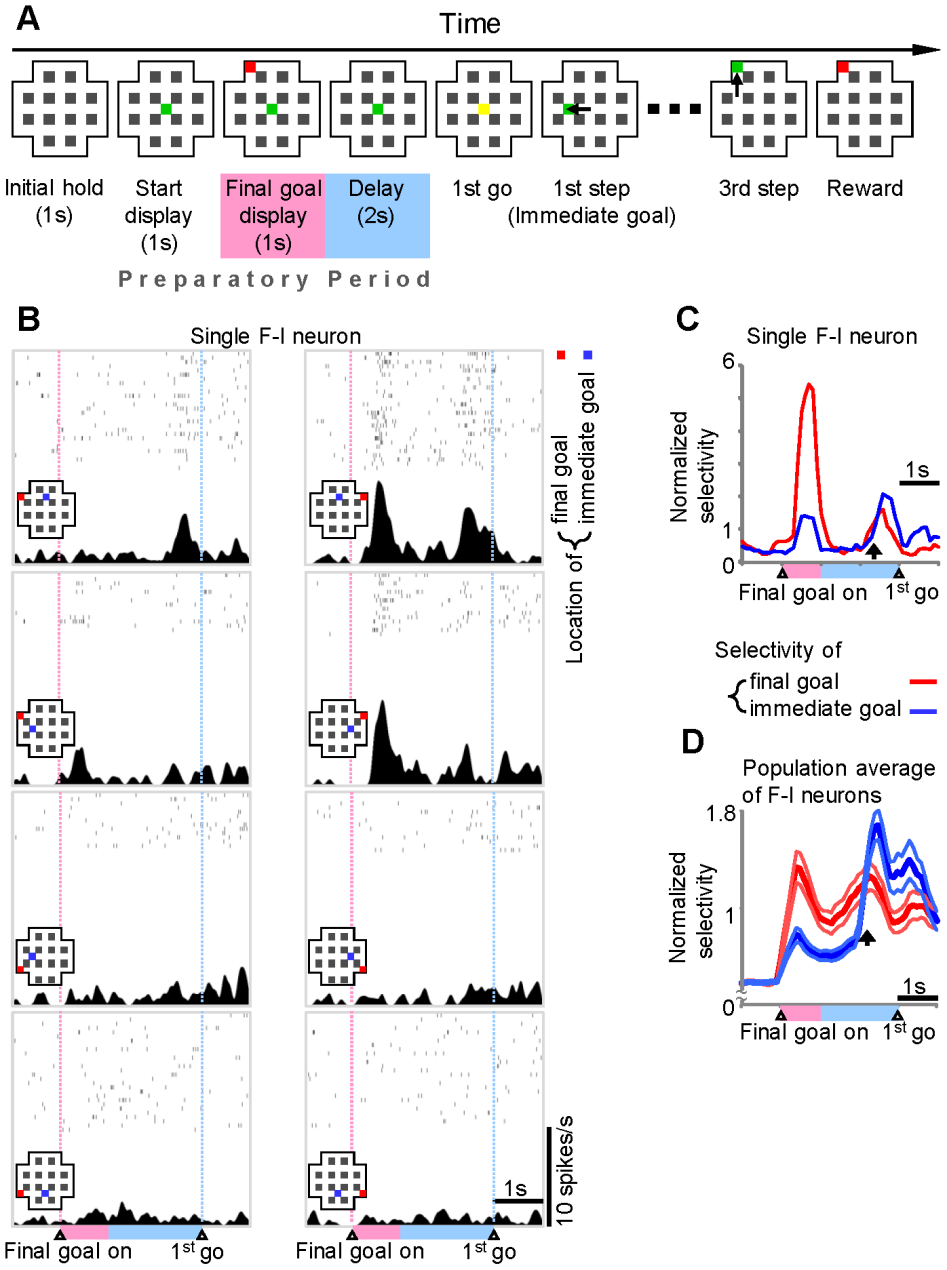
lPFC neurons showing representational transitions. (A) Temporal sequence of events in the path-planning task (maze task). The behavioral sequence is depicted from left to right. Each panel represents a maze displayed on a monitor, with green squares denoting current cursor positions, and red squares representing the position of the final goal. Yellow squares represent movement initiation (go) signals, and black arrows delineate cursor movements. Start display, final goal display, and delay periods constitute the preparatory period. (B) Discharge properties of an lPFC neuron that represents the final goal followed by the immediate goal during the preparatory period. Raster plots and spike-density histograms of neuronal activity under task conditions for each combination of final and immediate goals are shown. A red square indicates the location of the final goal remembered during the preparatory period, and a blue square indicates the planned immediate goal. In the early phase of the preparatory period, this neuron was selectively active when the final goal was located at the top right of the maze. In the late phase, selectivity was most prominent when the immediate goal was above the starting position. (C) The time course of modulation of the final- (red line) and immediate-goal (blue line) selectivity of the neuron shown in B. The goal selectivity, or regression coefficient, is normalized by the *t* value at the significant level, *P* = 0.05. (D) The mean ± SEM of selectivity for the final (red line) and immediate (blue line) goals of the population of neurons (*n* = 148) with F-I (final-immediate) shifts. Arrows, F-I transition times.

These monkeys were trained on the path-planning task (maze task) as previously reported [4], [21]–[23] (Fig. 2A). The monkey was required to move a cursor step by step to reach a final goal in a checkerboard-like maze on a monitor. After 1 s (Initial hold), a green cursor appeared at the center of the maze on a monitor (Start display), and 1 s later, a red square was displayed for 1 s, indicating the position of a final goal (Final goal display). After a delay of 1 s, one or two of four possible paths to the goal were blocked in some trials. This was followed by another 1-s delay (Delay). Thereafter, when the cursor color was changed from green to yellow (1st go), the animal was required to move the cursor within 1 s to the first position (immediate goal). Then, the animal had to move the cursor stepwise to reach the final goal, where the animal was rewarded. Supination and pronation of each forearm were assigned to four cursor directions. To dissociate arm and cursor movements, the arm–cursor assignments were varied in three different combinations following completion of a block of 48 trials. In >89% of trials, the animals reached the goal within three movements of the cursor.

### Physiological Experiment and Analyses

Conventional electrophysiological techniques were used to obtain *in vivo* single-cell recordings [4], [22], [23] from the lateral prefrontal cortex (lPFC) above and below the principal sulcus in the right hemisphere. Cortical sulci were also identified using a magnetic resonance imaging scanner (OPART 3D-System; TOSHIBA). Eye position was monitored using an infrared eye-camera system (R21–C–AC; RMS). Neuronal activity was not associated with eye position or eye movement. Individual spikes were isolated using a template-based discriminator (Multi-Spike detector; Alpha-Omega). Only well-isolated spikes that were stable over entire recordings and had clear single peaks in the distribution of distance from the template were included in the analysis.

This study focused on neuronal activities during the preparatory period (Start display, Final goal display, Delay). To statistically assess how the final and immediate goals were related to cell activity, a linear regression analysis [24] was conducted using the following regression model: firing rate = *β*_0_+ *β*_1_ × (final goals)+*β*_2_ × (immediate goals), where *β*_0_ is the intercept, and *β*_1_ and *β*_2_ are the regression coefficients. The categorical factors for final and immediate goals were horizontal and vertical directions. The firing rate was calculated as spike counts in 100 ms. The time development of the coefficients was normalized by the significance level of the *t*-value (*P*<0.05).

After the time evolution of the final goal (*FGS*[*t*]) and the immediate goal selectivity (*IGS*[*t*])) were obtained, the *F-I index* (final goal-immediate goal index) was calculated as *F-I index* (*t*) = [*IGS*(*t*) – *FGS*(*t*)]/[*IGS*(*t*)+*FGS*(*t*)]. Neurons that showed representational shifts from final to immediate goals were defined as F-I neurons (final goal-immediate goal neurons) whose *F-I index* showed a negative-to-positive change and, at its maximum value, the *IGS* was significant [4]. We also defined neurons that exhibited significant selectivity for the final, but not immediate, goals as final-goal neurons.

The duration of extracellular spike waveforms was also analyzed to classify neurons as putative pyramidal neurons or interneurons [25]–[27]. Two time distances from each waveform were obtained, one between the trough and the peak and the other between the inflection point marking the beginning of the initial negativity and the return to baseline after the first positive deflection. Dots for each waveform were plotted on the two-dimensional space of the two distances, and the norms from the origin provided a consistent classification of putative inhibitory and excitatory neurons.

### Evaluation of Firing Variability

To assess firing variability, variability in interspike intervals (*ISI*) was evaluated using measures developed to eliminate the influence of firing rate [28]–[31]. Unless otherwise noted, the firing variability was evaluated by *L_V_R* [31]. A constant, *R*, which compensates for the refractoriness effect of a previous spike, was introduced to exclude the influences of firing rate. The mean *L_V_R* was defined as follows:
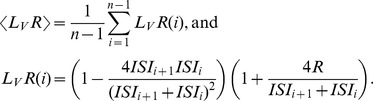



*ISI*s were calculated with a time resolution of 1 ms, and *n* is the number of *ISI*s during the period of interest. For simplicity, <*L_V_R*> is referred to as *L_V_R*. The influence of the firing rate was successfully excluded by using *L_V_R* (*R* >10 ms). Here, we used *R* = 11 ms.

Other measures, including the local variance *L_V_* [28], were used as well:
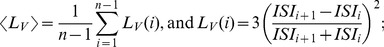



*IR* [29],
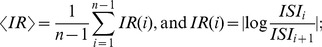
and *SI* [30],



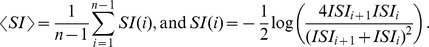



These parameters were measured for each 100 ms epoch during the preparatory period.

Note that the focus of this study was restricted to the task-dependent modulation of firing variability rather than its absolute value.

### Neural-network Models

Here, the dynamical state of neural networks [3], [9], [32] consisting of two mutually connected populations *X*_1_ and *X*_2_ were considered. The dynamics of each is described as follows:
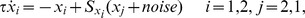
where *x_i_* was the activity of node *X_i_*, and *τ* is the time constant (20 ms) [17], [18]. S*_xi_*(*x_j_*) was the gain function from populations *X_j_* to *X_i_*. The following first order Naka-Rushton function was used [33]–[36] where the output was limited between 0 and 1:



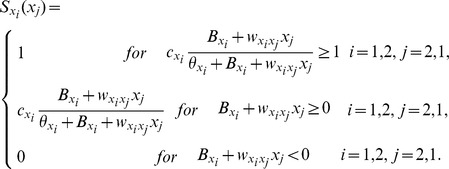
Here, *c_xi_*, *B_xi_*, and *θ_xi_* define the maximum effect of input, the offset, and the value of *x_i_* at which *S_xi_*(*x_j_*) reaches the half of the maximum, respectively. By varying these parameters, the shape of the gain function could be controlled systematically. 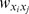 is the connectivity from population *x_j_* to *x_i_*; its value is 1.0 for excitatory and −1.0 for inhibitory connectivity. As the source for fluctuations in the population activities, low levels of Gaussian noise (σ = 0.025 or 0.01) were added to the gain functions at each time step [3], [17], [18]. The fluctuations of population activities will be diminished or amplified depending on the stability of point attractors in the networks.

For these population activities to reflect the firing rate of a neuron directly, a phase model was used in which the activity of the population defined the phase velocity as follows [35], [37]:
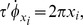
where *τ*’ is the time constant (50 ms), and the neuron fires when the phase *φ* reaches an integer multiple of 2π. The neuron fired when the phase *φ* reached an integer multiple of 2π. The maximum population activity corresponds to 20 spikes/sec.

The differential equations were simulated by the Runge-Kutta method with the time step *Δt* = 0.05 ms. Each calculation was done for 60,000 steps and repeated 100 times. Each parameter is described in Text S1. The code corresponding to these implementations is provided in the ModelDB database (https://senselab.med.yale.edu/modeldb/ShowModel.asp?model=151127).

### The Stability of Point Attractors

For the cases of two-node networks, the dynamics of the deviations *Δx_1_* and *Δx_2_* around a point attractor (*x_1_0_*, *x_2_0_*) in the network of two mutually connected populations *X*_1_ and *X*_2_ is approximated as follows (Fig. S1A):
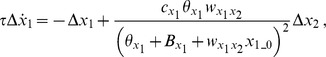


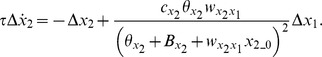



The maximum Lyapunov exponent (MLE) is defined as the maximum real part of eigenvalues of the Jacobian matrix for these linearized differential equations. The MLE for the above equations can be represented as
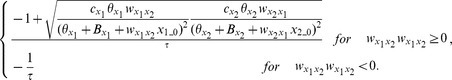



If the network is excitation–inhibition, the MLE stays constant at −1/τ. By varying the gain function of each node, the MLE was systematically controlled.

### “Stiffness” as the Second Stability Index

Here, another index for the stability of point attractors referred to as “stiffness” was introduced. Τhis corresponds to the stiffness coefficient in a spring pendulum model represented by a one-dimensional second-order linear differential equation (Fig. S1B). Using this index, it is possible to assess the stability of point attractors in excitation–inhibition networks whose stability cannot be assessed by the MLE. The generalization of this index to *n*-dimensional systems is also discussed.

### “Stiffness” in Two Dimensional Systems

The stability of a steady state in a dynamical system is usually discussed in relation to its linear approximation of the small deviation from the steady state (Fig. S1A). The MLE is defined as the maximum real part of the eigenvalues of the Jacobian matrix for the linearized differential equations. This has been used as a standard index for the stability of an attractor for perturbations. However, influences of the imaginary parts of eigenvalues on the stability are beyond the scope of the MLE. For this reason, MLEs are not suitable for quantification of the stability of excitation–inhibition networks, because the eigenvalues for a point attractor of an excitation–inhibition network inevitably includes imaginary parts. Thus, an index called “stiffness” was considered. In the case of two mutually connected neural populations *X_1_* and *X_2_* in the main text, the time evolution of the small deviations Δ*x_i_* (*i* = 1, 2) of their activities *x_i_* can be expressed as follows:
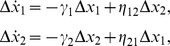
where *γ_i_* and *η_ij_* (*i* = 1, 2; *j* = 2, 1) are decay factors that were fixed to −1 in all of the calculations, and connection coefficients, respectively. These two-dimensional first-order linear differential equations can be transformed into a one-dimensional second-order differential equation as follows:



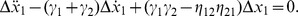



Here we compare this equation with a spring pendulum (Fig. S1B) that is described by the following one-dimensional second-order linear differential equation:
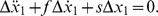



The coefficients *f* and *s* can be regarded as a friction coefficient and a stiffness coefficient, respectively. For this spring pendulum, a potential can be defined using this stiffness coefficient as follows:
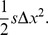



A larger stiffness coefficient provides a deeper potential. Therefore, the spring pendulum is more attracted to the singular point for a certain deviation. Consequently, for an identical perturbation to the system, a system with a deep potential is less sensitive to it than that with shallow potential (schematized in Fig. S1C). Thus, “stiffness” is defined as
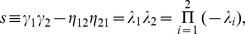
where *λ_i_* is an eigenvalue of the system (*i* = 1, 2). Note that this index includes the influences of the imaginary parts of eigenvalues. Here, it is assumed that all eigenvalues are negative because point attractors are considered in this argument. Thus, the stiffness for the point attractor for the two-node networks is described as follows:



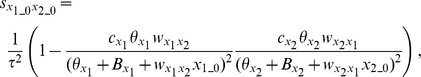
where *x_i_*, *τ*, *c_xi_*, *B_xi_*, *θ_xi_* and *w_xixj_* define the activity of node *X_i_*, the time constant, the maximum effect of input, the bias, the value of *x_i_* at which the gain function reaches a half of the maximum, and the connectivity from population *X_j_* to *X_i_*, respectively.

### Generalization of “Stiffness” to n-dimensional Systems

The definition of stiffness can be easily extended to higher-order dynamical systems and can be generalized for networks that include *n* mutually connected populations as follows:
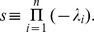
where *λ_i_* is an eigenvalue of the system (*i* = 1, 2, … *n*). Again, it is assumed that all eigenvalues are negative. The *n*-dimensional coordinates *x_i_* (*i* = 1, …, *n*) in which the activities of the *n* populations are represented can be transformed into the other coordinates *x’_i_* (*i* = 1, …, *n*), each of which is defined as the direction of each eigenvector. By using these new coordinates, the potential can be defined as



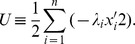



Then, the volume of hyper-ellipsoid surrounded by the equipotential surface of *U* = *U*_0_ is
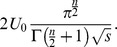



Γ is a gamma function. This means that as *s* is larger, the volume of the hyper-ellipsoid becomes smaller. That is, the larger *s* is, the deeper the potential becomes.

Another advantage of the generalized stiffness is that it can be easily obtained for higher-order dynamical systems by considering the relationship between solutions and coefficients in the Jacobian determinant without solving it, that is, from the constant term of the characteristic polynomial for arbitrary-dimensional systems.

## Results

Of the 887 neurons whose activity was recorded from the lateral prefrontal cortex (lPFC) while monkeys were performing a maze task (path-planning task) (Fig. 2A), we found 148 F-I neurons (final goal-immediate goal neurons) that exhibited representational shifts in behavioral goals coded by the firing rate during the preparatory period. We also obtained 259 final-goal neurons that exhibited significant selectivity for the final, but not immediate, goals during the same period.

An example of lPFC neurons that exhibited an F-I transition is shown in Fig. 2B. During the early phase of the preparatory period, the firing rate increased selectively when the final goal was located in the top right quadrant of the computer screen. In the late phase of the preparatory period, the firing rate was highest when the animals had planned on the immediate goal being located above the start position. To visualize the time course of the representations of this cell for the final and immediate goals, we plotted the goal-selectivity determined by regression analysis for consecutive 100 ms time frames, as described in the Materials and Methods (Fig. 2C). The results show how the final goal representation was developed, reduced, and then replaced with the immediate goal representation. This temporal pattern was also confirmed by population analysis of F-I neurons (Fig. 2D). In contrast, population analysis of goal selectivity of final-goal neurons revealed almost constant selectivity for the final goals throughout the preparatory period (Fig. S2). This suggests that these neurons were involved in spatial working memory for the position of the final goals, which has long been observed in the lPFC.

**Figure 3:**
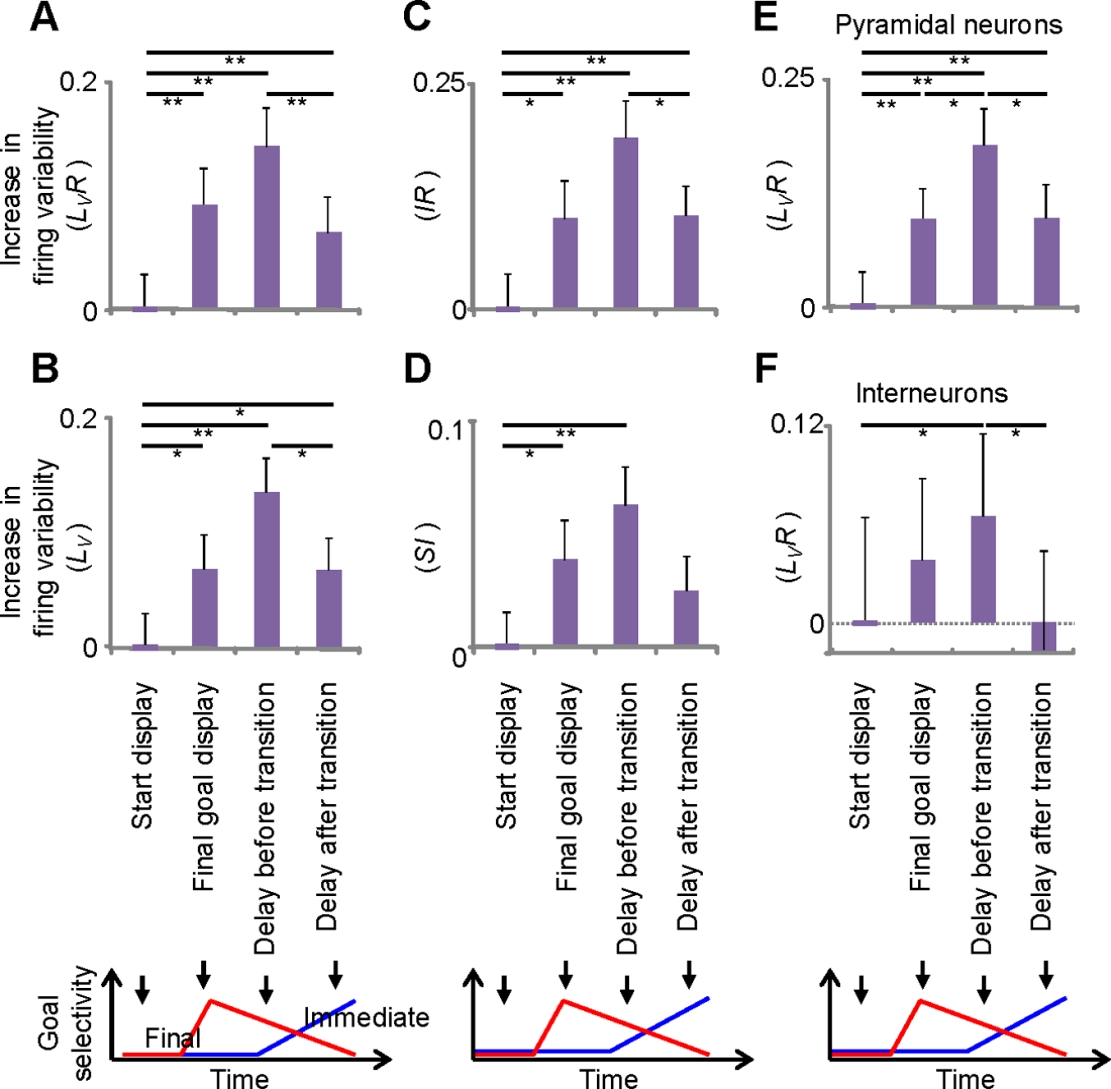
The firing variability of F-I neurons increases before the representational transitions. (A) The average *L_V_R* increases in three epochs (final goal display, delay before transitions, and delay after transitions) from the initial value (start display; 1.11) (*n* = 148). (B–D) Increases from the initial values of firing variability: *L_V_*, 0.88 (B); *SI*, 0.28 (C); *IR*, 1.21 (D). (E and F) Increases in the firing variability of the putative excitatory (*n* = 110; E) and inhibitory neurons (*n* = 38; F). The initial values are 1.12 and 1.08, respectively. Start display, −700 to −800 ms; final goal display, 400 to 500 ms; delay before transitions, 1100 to 1200 ms from the final-goal onset; delay after transitions, 200 to 300 ms after F-I transition. Error bars = SEM; *, *P*<0.05; **, *P*<0.01 (*t*-test).

To assess the idea that the representational shifts could be considered state transitions in the underlying neural network, the firing irregularity in F-I neurons of lPFC was analyzed. As mentioned above, lPFC neurons exhibit task-dependent firing-rate modulation. The use of indices that are robust against the influences of such modulations can be used to evaluate firing irregularity. Using *LvR* [31], we could successfully exclude the influence of firing rate (*r* = 0.026, *P*>0.05) [38]. Figure 3A shows the changes in *LvR* for four epochs: start display, final goal display, delay before transition, and delay after transition. F-I neurons exhibited gradual increases in firing variability, and reached a maximum value in the delay before the transition epoch, which was significantly higher than the reference value obtained in the start display epoch (*P*<0.01, *t*-test), whereas the firing rates of these two epochs were comparable (5.7 spikes/s). More importantly, the firing variability during the delay before the transition epoch was reduced significantly in the delay after the transition epoch (*P*<0.01, *t*-test; Fig. 3A). This profile of firing variability in F-I neurons contrasted with the final-goal neurons (Fig. S3). Consistent with previous reports [39], [40], these neurons exhibited an increase in firing variability during the delay period compared to baseline (start display) (*P*<0.01, *t*-test). However, there was no significant decrease in firing variability in the epoch corresponding to delay after transition in F-I neurons (*P* = 0.47, *t*-test). In addition, the values of firing variability in this epoch were significantly different between F-I and final-goal neurons (*P*<0.01, *t*-test). Similar temporal patterns were observed using other indices that are unaffected by firing-rate modulation (Fig. 3B–D).

Cortical neurons are subdivided into excitatory pyramidal neurons and inhibitory interneurons. To determine whether the temporal pattern of firing variability was dependent on neuronal type, F-I neurons were classified into two groups [25]–[27]. Both putative excitatory (*n* = 110) and inhibitory (*n* = 38) neurons exhibited significant increases in firing variability prior to the representational shifts (*P*<0.05, *t*-test). These analyses support the hypothesis that firing variability in lPFC neurons increases with the representational shifts, regardless of neuronal type (Fig. 3E, F).

These results strongly suggest that the representational shifts in behavioral goals reflect state transitions in the underlying neural network. However, it is unknown whether these increases in firing variability are caused by a destabilization of the network. Therefore, to investigate how variability in spike trains is influenced by the stability of dynamical systems in the network, a simple computational neural-network model composed of mutually connected neural populations was used. Each neuron belonged to a population and emitted spikes dependent upon the activity of the population. By controlling the parameters of the gain functions in the neural populations, the degree of network stability was systematically modulated. To examine how firing variability is influenced by the vulnerability of network to perturbations, constant Gaussian noise was added to the network. This model allowed for examination of the relationship between the stability of the neural network and firing variability (see materials and methods).

**Figure 4:**
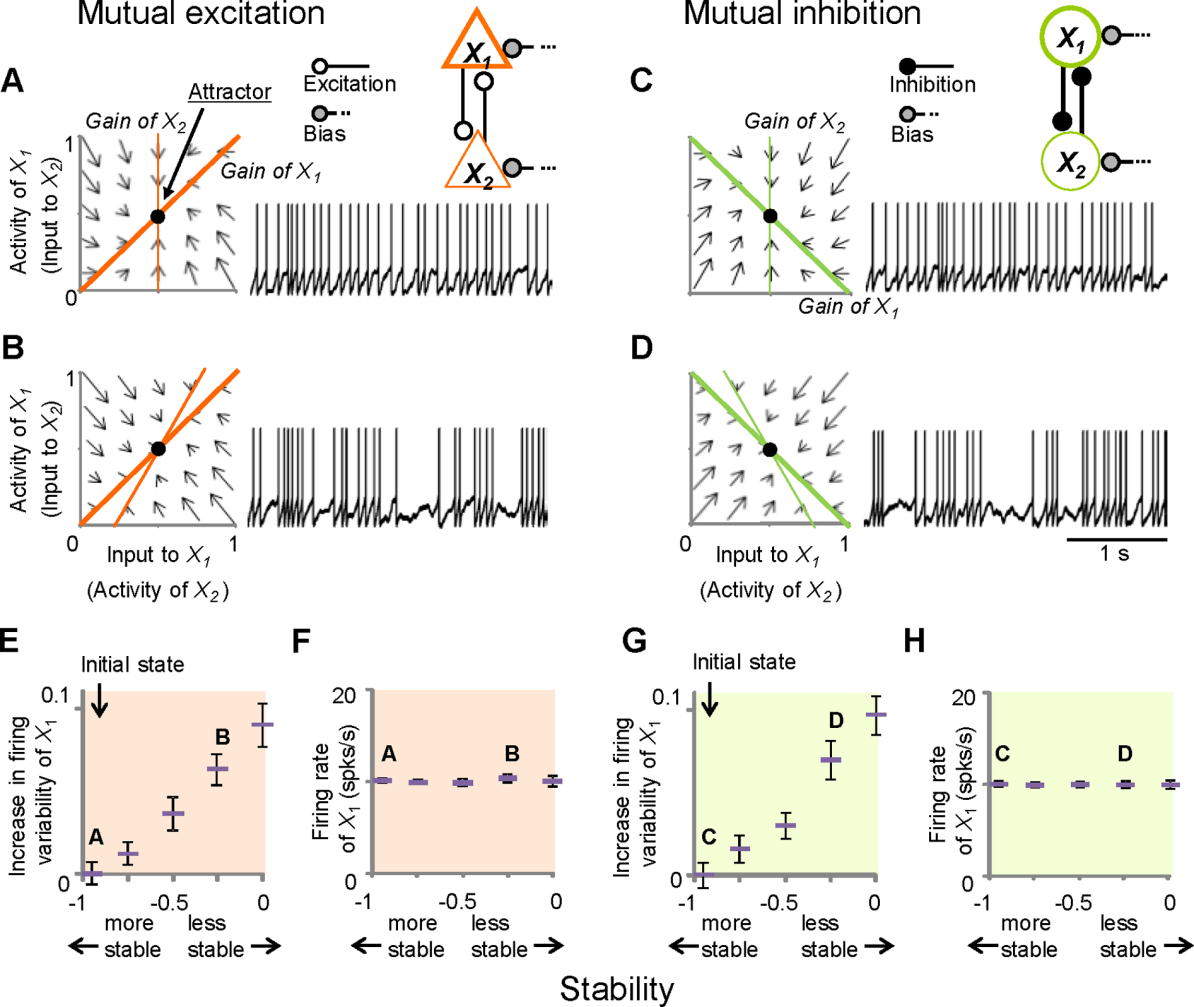
Neural network models show changes in firing variability with stability. (A and B) Phase-plane plots (left) for a mutual-excitation circuit and firing of a neuron in node *X*_1_ (right). Each node represents a population of neurons. The thick and thin orange lines in the plots are gain functions for *X*_1_ and *X*_2_, respectively. Arrows represent vector fields, and black circles delineate point attractors. (C and D) Mutual inhibition is presented by green lines, and represents gain functions. In these phase plane plots, these gain functions denote null clines, where 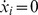 (*i* = 1, 2). (E and G) Increases in the firing variability of the *X*_1_ neuron with the maximum Lyapunov exponent (MLE) from the initial states. The corresponding firing rates (F and H), mutual excitation (E and F), and mutual inhibition (G and H) are presented. Error bars = SEM.

The present study primarily focused on simple networks in which two nodes of neural populations were mutually connected (mutual excitation, Fig. 4A, B; mutual inhibition, Fig. 4C, D; excitation–inhibition, Fig. S4A–D). To graphically understand the interaction between two mutually connected nodes, the input–output relationship, or nullcline, was plotted in a two-dimensional phase plane. In these plots, the two input–output functions or gain functions are superimposed, with the activity of *X*_1_ as a function of the input from *X*_2_ (thick lines); the gain function of *X*_2_ can be plotted by exchanging the horizontal and vertical axes (thin lines). The points where the two gain functions intersect are referred to as equilibrium points or fixed points. If the states of the systems converge onto an equilibrium point with time, the points are referred to as point attractors (black dots).

Variability in neuronal firing was influenced by the gain functions of the population to which the neuron belonged, and the other populations in the neural network. An example of a mutual-excitation network is shown in Fig. 4A and B. In these cases, making the gain function of node *X*_2_ steeper resulted in increased neuronal firing variability in both *X*_1_ and *X*_2_ when keeping the gain function of *X*_1_ fixed. This was true in cases of mutual-inhibition networks (Fig. 4C, D). Thus, if the gain function of node *X*_2_ became steeper, the firing variability in both *X*_1_ and *X*_2_ increased. In excitation-inhibition networks, changing the gain functions caused changes in firing variability (Fig. S4A–D). Interestingly, however, the firing variability of *X*_1_ decreased even if the gain function of node *X*_2_ got steeper. These calculations suggest that changes in firing variability should be considered dynamic properties on the network level, particularly the stability of point attractors.

To quantify the stability of the networks, the maximum Lyapunov exponent (MLE) was used as an index reflecting the degree of convergence speed to an attractor. When MLE is negative, the point attractor is stable because the system is able to return to the attractor from small perturbations. To assess the relationship between the stability of point attractors and firing variability, MLE values were systematically controlled by selecting the appropriate parameters of gain functions in *X*_1_ and *X*_2_. Neurons exhibited systematic increases in firing variability as the point attractor became less stable, as indicated by observations that the MLE was approaching zero in both the mutual-excitation and mutual-inhibition networks (Fig. 4E, G). These changes were not associated with changes in firing rates (Fig. 4F, H). The mean firing variability and firing rate of the neurons shown in Fig. 4A–D are presented in Fig. 4E–H.

These findings also demonstrated that systematic changes in firing variability were dependent on the stability of point attractors in the excitation-inhibition networks (Fig. S4E, G) without changing firing rates systematically (Fig. S4F, H). In these calculations, however, we evaluated the stability of the network point attractor with “stiffness” introduced instead of MLE, because excitation-inhibition networks inevitably include an oscillatory component. If the networks do not include an oscillatory component as mutual excitation or inhibition networks, stiffness can provide results that are consistent with MLE (Fig. S5). The simulation data showed that the firing variability increased systematically as stiffness decreased (Figs. S4E, G and S5).

We also demonstrated that the firing variability increased systematically with the attractor stability of the network in which three nodes were interconnected (Fig. S6). Importantly, firing irregularity increased systematically as stiffness decreased in three node networks, even if some connections in the networks changed from inhibition to excitation. Based on these data, we concluded that the stability of point attractors in neural networks affect the firing variability of the neurons.

**Figure 5:**
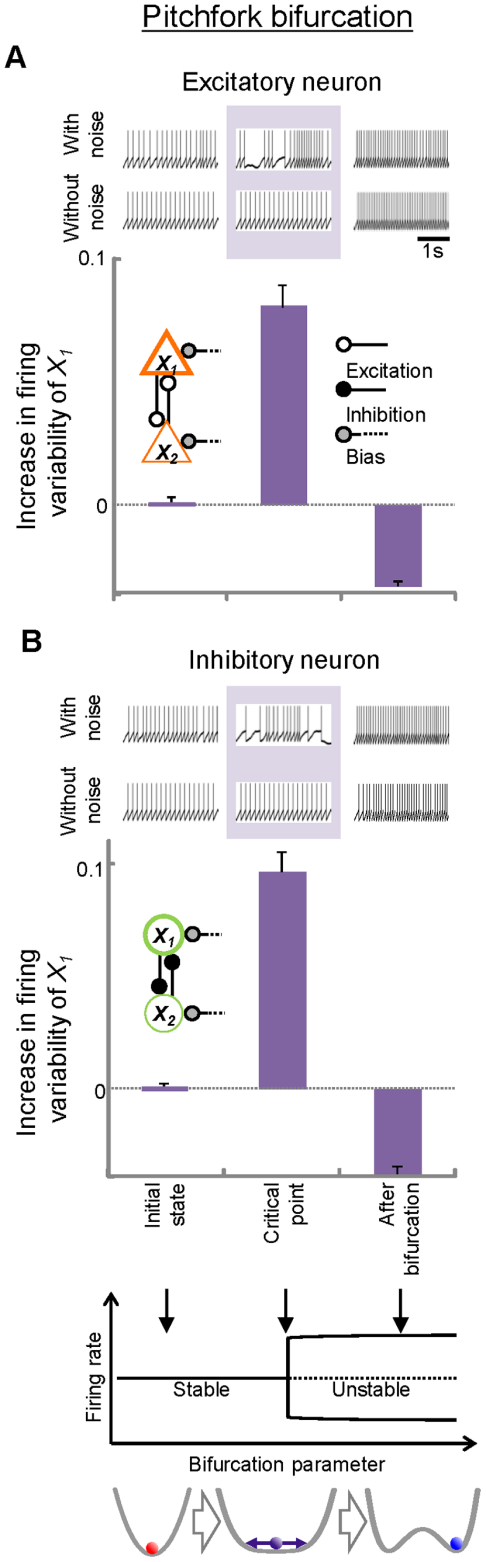
Changes in firing variability before and after transitions in neural- network models showing pitchfork bifurcation. Increases in *L_V_R* from the initial values are plotted for both excitatory (A) and inhibitory (B) neurons. Schematic illustrations for pitchfork bifurcation are indicated at the bottom: solid lines: stable attractors; dotted lines: unstable saddles. Examples of firing for each case are shown. Also shown are corresponding firing patterns obtained under the without-noise conditions for comparison. Error bars, SEM.

**Figure 6:**
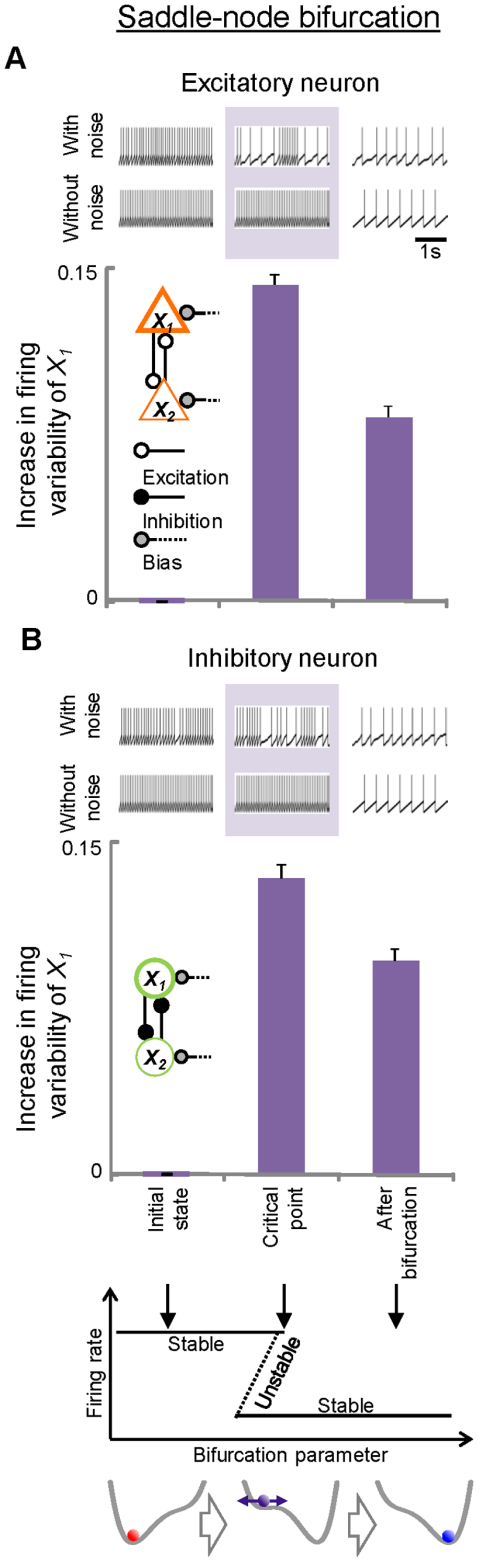
Changes in firing variability before and after transitions in neural- network models showing saddle-node bifurcation. Increases in *L_V_R* from the initial values are plotted for both excitatory (A) and inhibitory (B) neurons. Schematic illustrations for saddle-node bifurcation are indicated at the bottom: solid lines: stable attractors; dotted lines: unstable saddles. Examples of firing for each case are shown. Also shown are corresponding firing patterns obtained under the without-noise conditions for comparison. Error bars, SEM.

**Figure 7:**
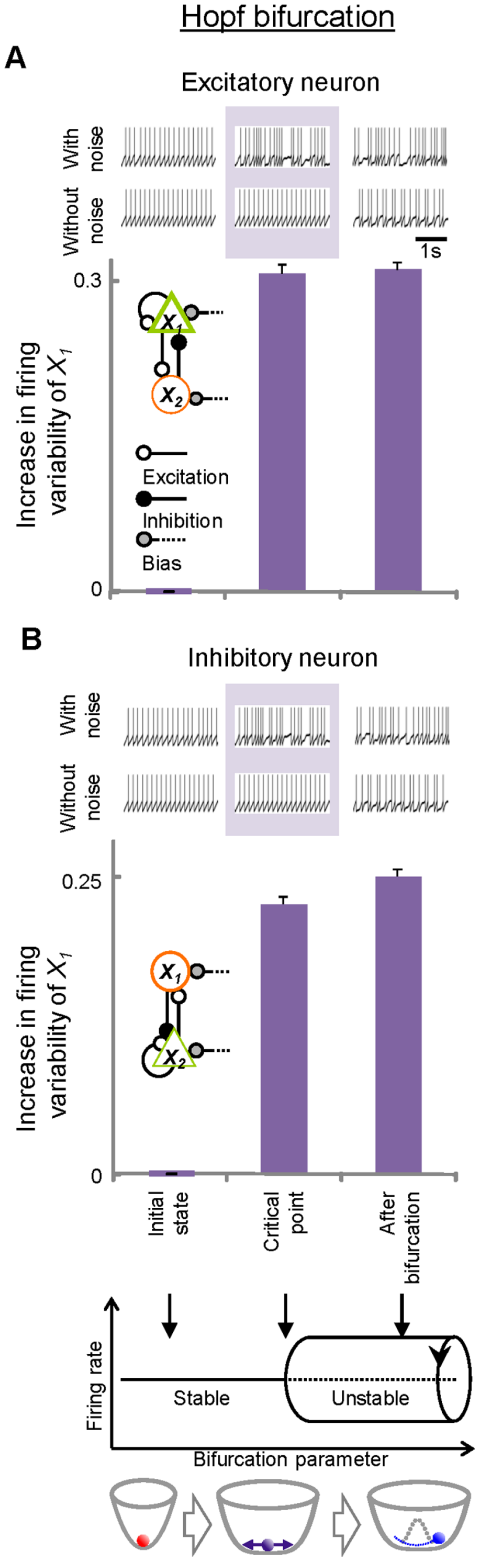
Changes in firing variability before and after transitions in neural- network models showing Hopf bifurcation. Increases in *L_V_R* from the initial values are plotted for both excitatory (A) and inhibitory (B) neurons. Schematic illustrations for Hopf bifurcation are indicated at the bottom: solid lines: stable attractors; dotted lines: unstable repellers. Examples of firing for each case are shown. Also shown are corresponding firing patterns obtained under the without-noise conditions for comparison. Error bars, SEM.

Next, to assess the direct relationship between firing variability and state transitions of neuronal networks, firing variability was evaluated in the major types of bifurcations (pitchfork [Fig. 5], saddle-node [Fig. 6], and Hopf bifurcations [Fig. 7]) by changing parameters systematically across the critical points of the bifurcations. In each bifurcation, increases in the firing variability of excitatory (Figs. 5A, 6A, 7A) and inhibitory (Figs. 5B, 6B, 7B) neurons were observed when the systems were approaching bifurcations at critical points compared to the initial states. At these critical points, instability in the networks manifested as increases in firing variability only when noise was added to the networks (firing patterns in pale purple areas, Figs. 5, 6, 7). These data suggest that the networks become vulnerable to a constant level of perturbations at critical points, and that the vulnerability is reflected in firing variability. After the bifurcation, the firing variability depends on the type of bifurcation that occurred. In pitchfork and saddle-node bifurcations, the states of the networks shifted or jumped to another point attractor, resulting in decreased firing variability. In contrast, the firing variability remained high after Hopf bifurcation because the point attractors became unstable with oscillatory activities.

## Discussion

We assessed the hypothesis that increases in firing irregularity are a crucial measure for predicting state transitions in the underlying neuronal circuits in the prefrontal cortex. Experimentally, we analyzed the activities of neurons in the prefrontal cortex while monkeys performed a maze task that required them to perform actions to reach a goal. We identified increases in the firing variability of F-I neurons in the lPFC as an emergent property of state transitions in which the neuronal representation shifted from the final goals of behavior to action. Then, we constructed theoretical generic neural networks and demonstrated that changes in neuronal gain on functional connectivity caused a loss of their stability and altered the state of circuits, resulting in increased firing irregularity. The network-dependent irregularity was a robust phenomenon for the major classes of bifurcations or state transitions in dynamical systems, regardless of the type of neuron (excitatory or inhibitory) or network configuration (mutual excitation, mutual inhibition, or excitation-inhibition). Therefore, this suggests that increases in neuronal firing variability reflect the approaching of critical points for state transitions, with a loss of stability at a state of equilibrium in the network.

### Firing Irregularity in the Prefrontal Cortex from the Viewpoint of Dynamical Systems Theory

We identified two types of neurons in the prefrontal cortex: F-I neurons with representational changes, and final-goal neurons with sustained activity reflecting the final goal. From the dynamical systems view, a transient increase in the irregularity of F-I neurons reflected instability at a critical transition, as predicted from the behavior of our model network. Nevertheless, how to interpret the sustained irregularity of goal-related neurons appropriately must be considered. If sustained activity represents a stable, active state of bistability of the network, there should be little firing irregularity, similar to the stable resting state. Instead, tonic irregularity during sustained activity seems to reflect tonic instability of the network, which reflects the active holding of information in the working memory. Consistent with this, Compte et al. [39] observed that the prefrontal neurons showed increased firing variability in the delay period of working memory tasks. Nevertheless, understanding the increased firing variability and stable retention of working memory comprehensively is challenging [41], [42]. Machens et al. [3] reported parametric working memory in the prefrontal cortex during a vibration comparison task, and proposed a dynamical network model that held information with a line attractor network with less stability. In their model, working memory reflected the accumulation of evidence for future decision-making required for the task. However, working memory is not only used to maintain information in the short term, but also for processing information in the executive function of the prefrontal cortex. According to Baddely’s working memory model, the central executive, which acts as a supervisory system, controls the flow of information using the working memory as a “slave system” [43]. Therefore, sustained activity could be considered a pending state of the network near the critical point, open for further phase transitions in a flexible manner for updating neural representations, such as decision-making and planning. In the present study, information on the final goal could be used at any time to update action plans to achieve the final goal. Based on our current findings and the dynamical systems theory, a transient and tonic increase in firing irregularity of the prefrontal cortex reflects two aspects of executive function: stable maintenance of information, and flexible updating of information flow. This is consistent with the idea that the prefrontal cortex, as the central executive, controls information flow [44]–[46].

### Circular Interactions between Local Gain and the Global State of the Network

We found that changes in the stability of attractors and bifurcations at the network level could be induced by modulating gain functions at the level of neuron or synapse (Fig. S7A). In addition, the stabilization of the attractor at the level of the network or representation affected firing variability (Fig. S7B). Recent studies have indicated that firing variability or spiking noise could modulate the gain function, particularly the slope and offset at the level of the neuron or synapse [47]–[50] (Fig. S7C). Therefore, gain and stability interact across hierarchies between the levels of the network and neuron/synapse via firing variability. Indeed, local changes in connectivity can induce a global network state, and vice versa. The mutual dependence of gain and stability suggest that the prefrontal cortex is a self-organizing dynamic system [51]. Therefore, the network is able to remain far from a state of equilibrium and evolve towards an emergent network state depending on balance between the stability of attractors and the flexibility of bifurcations. Metaphorically, this relationship between flexibility and stability could be described as the yin-yang concept, in which seemingly opposite or contradictory forces interrelate to each other to form a dynamic system beyond the sum of its parts. Because of this relationship, the system tends to stay at a less stable attractor for a while accompanied by fluctuations.

### Limitations and Generalization of Network Models

The computational model used in this study is highly simplified. However, it holds substantial generality for networks with large populations of neurons as discussed below. Biological systems including the nervous system are dissipative systems that operate out of, and often far from, points of equilibrium [52]. The dissipative system commonly involves a self-organization process, where global order or coordination results from local interactions. Although such systems generally have large degrees of freedom, the levels of many parameters can be converged rapidly to a steady state, resulting in an enormous reduction in degrees of freedom of the system. Therefore, the macroscopic behaviors of the systems, such as bifurcations, can be described approximately by a small set of less stable or unstable parameters, so-called order parameters [53]. Based on this, our analysis and discussion of a neuronal model with a relatively small number of parameters does not lose its basic generality. However, it should be noted that our system would lose its generality if the systems have other attractors, such as limit cycles or chaotic attractors. For example, networks with limiting cycles with noise resulted in irregular firings (Fig. 7). If the networks have chaotic attractors, the firing of neurons in the network will be irregular. Nevertheless, we propose that firing irregularity increases as the point attractors of the underlying neuronal networks become less stable.

### Schizophrenia as an Abnormal Meta-stability of a Network Losing Balance between Stability and Flexibility

Schizophrenia, one of the most debilitating mental illnesses, has been repeatedly associated with disturbances in the prefrontal cortex [54]. It results from an otherwise normal plasticity process during adolescence corresponding with the development of the prefrontal cortex [55]. Although schizophrenia remains poorly understood, working memory is a core cognitive deficit in schizophrenia due to primary deficits in the functioning of the prefrontal cortex [54], [56]. Rolls et al. [57], [58] proposed a dynamical systems scheme of schizophrenia in which the instability of high-firing-rate attractor states, which normally implement short-term memory and attention, contributes to the cognitive and negative symptoms of schizophrenia. Furthermore, noise-induced jumps to an attractor state with a higher firing rate, even in the absence of external inputs, contribute to the positive symptoms of schizophrenia. In contrast, Stephan et al. [59] proposed the disconnection theory of schizophrenia in which the core pathology of schizophrenia is impaired control of synaptic plasticity that manifests as abnormal functional integration of neural systems, i.e., dysconnectivity symptoms. Our data reveal important new information on both the instability and abnormal functional connectivity that underlie schizophrenia. Based on our scheme proposed above, the executive functions in the prefrontal cortex are critically dependent on the balance of stability and flexibility in metastable states with flexible functional connectivity. In this regard, schizophrenia could be characterized as a state of abnormal metastability with unstable flows of information. At the synaptic or genetic levels, small abnormalities of local networks may lead to disorders in the stability-gain interaction, and consequently result in an abnormal flow of information. At the macroscopic level, behavioral interactions with other individuals in psychological stress may induce multi-stable networks, and cause changes in the local gain in functional connectivity. In both cases, changes in the local gain and network states are amplified presumably in a self-organized manner, because of the circular interaction across hierarchies of network stability and gain of functional connectivity. This stability-gain interaction plays an important role in linking cognitive functions with network connectivity.

## Supporting Information

Figure S1 Stiffness as an index of the stability of dynamical systems. (A) A schematic view for linear approximations of input functions near a point attractor in the phase plane. (B) An image for a spring pendulum. (C) The stiffness coefficient, or the stiffness *s*, defines the deepness (or steepness) of the potential.
(TIF)

Figure S2 The lPFC neurons without showing representational transitions. The mean ± SEM selectivity for the final (red line) and immediate (blue) goals of the population of final-goal neurons (*n* = 259). The goal selectivity or regression coefficient is normalized to the significant level, *P* = 0.05.
(TIF)

Figure S3 Firing variability changes in final-goal neurons. The average *L_V_R* increases in three epochs (final goal display, delay before transition, and delay after transition) from the initial value (1.17) in the start display is shown (*n* = 259). Start display, −700 to −800 ms; final goal display, 400 to 500 ms; delay before transitions, 1100 to 1200 ms from the final-goal onset; delay after transitions, 200 to 300 ms after the mean F-I transition time of F-I neurons. Error bars = SEM; *, *P*<0.05; **, *P*<0.01 (*t*-test) for comparisons between epochs. †, *P*<0.01 (*t*-test) for comparisons between final-goal and F-I neurons.
(TIF)

Figure S4 Changes in firing variability in excitation–inhibition networks. (A and B) Examples of phase-plane plots (left) of the nullclines for an excitation–inhibition network and firing patterns of a neuron associated with the network (right). Each node represents a neural population. The thick green line and thin orange line in the phase-plane plots are nullclines for nodes *X*_1_ and *X*_2_ respectively. The grey arrows indicate the vector fields. Examples of neuronal firing are in node *X*_1_. Note that the gain functions of node *X*_1_ in A and B are identical, whereas those of *X*_2_ are changed. The value of 1 for the population activity corresponds to neuronal firing at 20 spikes/sec. (C and D), are the same figures for an inhibition–excitation network. The thick orange line and thin green line in the phase-plane plots are nullclines for nodes *X*_1_ and *X*_2_ respectively. (E–H) Systematic increases in firing variability from initial values (leftmost in E and G) with decreases in a stability measure “stiffness” (E and G) and without significant changes in firing rate (F and H). The firing variability of a neuron in *X*_2_ exhibited similar results. (E and F), Excitation–inhibition; (G and H), Inhibition–excitation. Black circles in the phase–plane plots represent stable equilibrium points (point attractors). Error bars, SEM.
(TIF)

Figure S5 Consistency between stiffness and the maximum Lyapunov exponents. (A) Increases in *LvR* with stiffness, *s*, for cases where *X_1_* received excitation from *X_2_*. Increases from the minimum value (*s* = 2.0) are plotted against the maximum Lyapunov exponent (MLE). Stiffness, *s*, was changed from 2.0 to 0.0 in 0.25 steps. The range of *s* from 2.0 to 21.0 corresponds to inhibition-excitation networks, and a re-plotting of the data in Fig. S4G. In this range of *s*, all MLEs were -1, because by definition they did not include the imaginary part of eigenvalues. In the range of *s* from 1.0 to 0.0 (where eigenvalues are not complex numbers, the networks are mutually excitatory, and the dynamics of networks do not include oscillatory components), stiffness and MLE have a one-to-one relationship. (B) As in (A) for cases where *X_1_* received inhibition from *X_2_*. For the range of *s* from 2.0 to 1.0, the data in Fig. S4E were re-plotted (excitation-inhibition). Note that the range of *s* from 1.0 to 0.0 corresponds to mutual inhibitory networks. These data are consistent with (A). Error bars denote SEM. Parameters for these calculations can be found in the supplementary information.
(TIF)

Figure S6 Changes in firing variability in three-node networks. The systematic increases in firing variability of a neuron in node *X*_1_ from the initial value (leftmost in A) with decreases in the stability measure “stiffness” are plotted. (A) Inhibition–excitation–excitation. (B) Mutual excitation. Parameters of input functions were set for the network with a point attractor at (0.5, 0.5, 0.5), so that a neuron emitted spikes at approximately 10 spikes/sec. The gain function of *X*_1_ was not changed in A and B, whereas those of *X*_2_ and *X*_3_ were changed and were identical. Note that these models exhibit systematic increases in firing variability as stability decreases (A and B) without significant changes in firing rate (C and D) across different network types, such as inhibition–excitation–excitation and mutual excitation. Error bars, SEM.
(TIF)

Figure S7 Proposed stability–gain interaction via noise. (A) Changes in neuronal gain functions determine the stability of the network and can cause bifurcations at the network level. (B) The state at the network level, particularly the stability of the attractor, can affect firing variability. (C) Firing variability can modulate the shape of the gain function determining the nullcline of the dynamics, in particular, its slope and offset, at the level of the neuron/synapse.
(TIF)

Text S1 Parameters of model calculations.
(DOC)
