# Supplementary figures and images for: Increased Firing Irregularity as an Emergent Property of Neural-State Transition in Monkey Prefrontal Cortex
Source: PLoS One. 2013 Dec 4;8(12):e80906. doi: 10.1371/journal.pone.0080906 (PMC3857743; doi:10.1371/journal.pone.0080906)

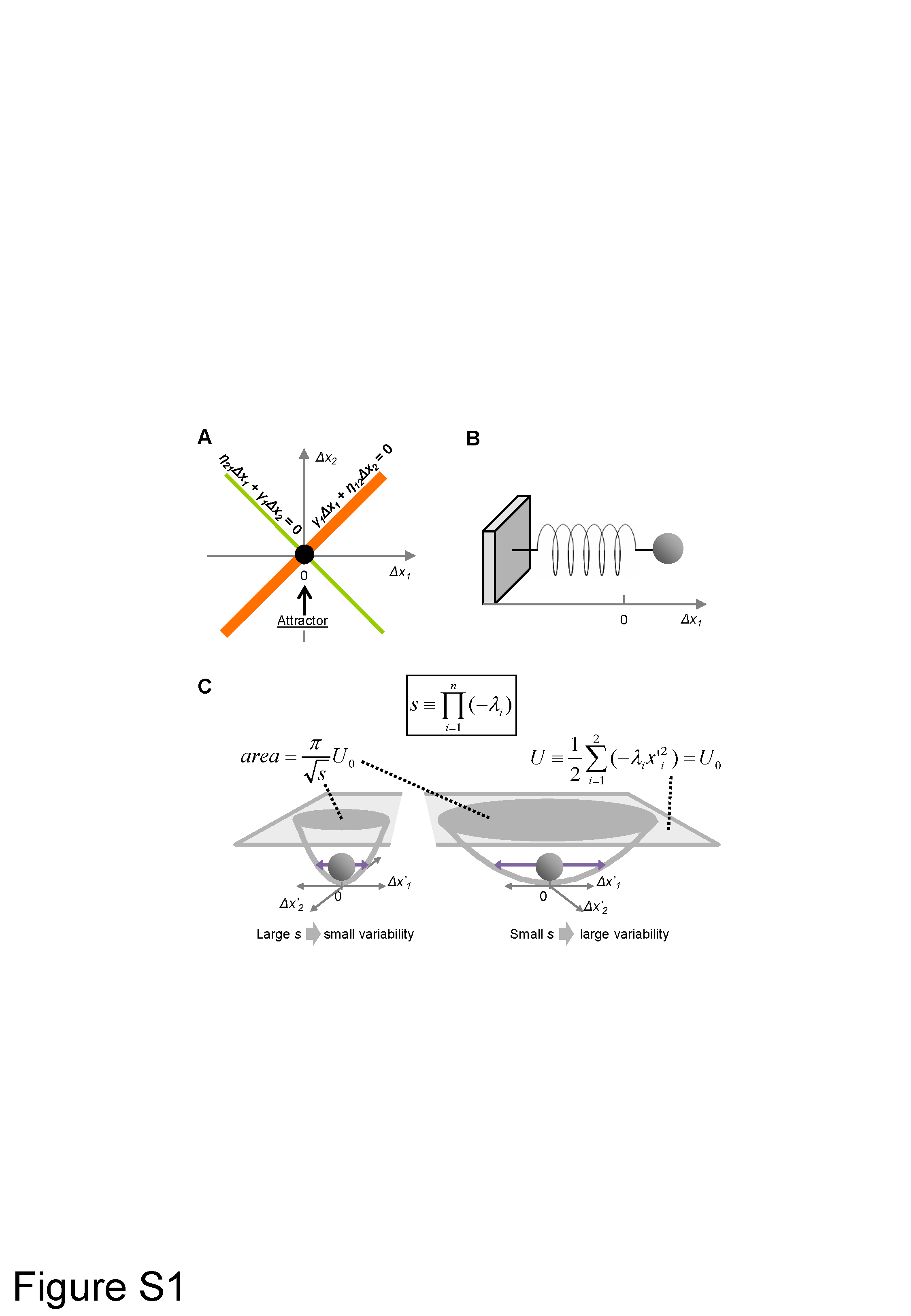

Supplement: Figure S1 — Stiffness as an index of the stability of dynamical systems. (A) A schematic view for linear approximations of input functions near a point attractor in the phase plane. (B) An image for a spring pendulum. (C) The stiffness coefficient, or the stiffness s, defines the deepness (or steepness) of the potential. (TIF) [file pone.0080906.s001.tif]

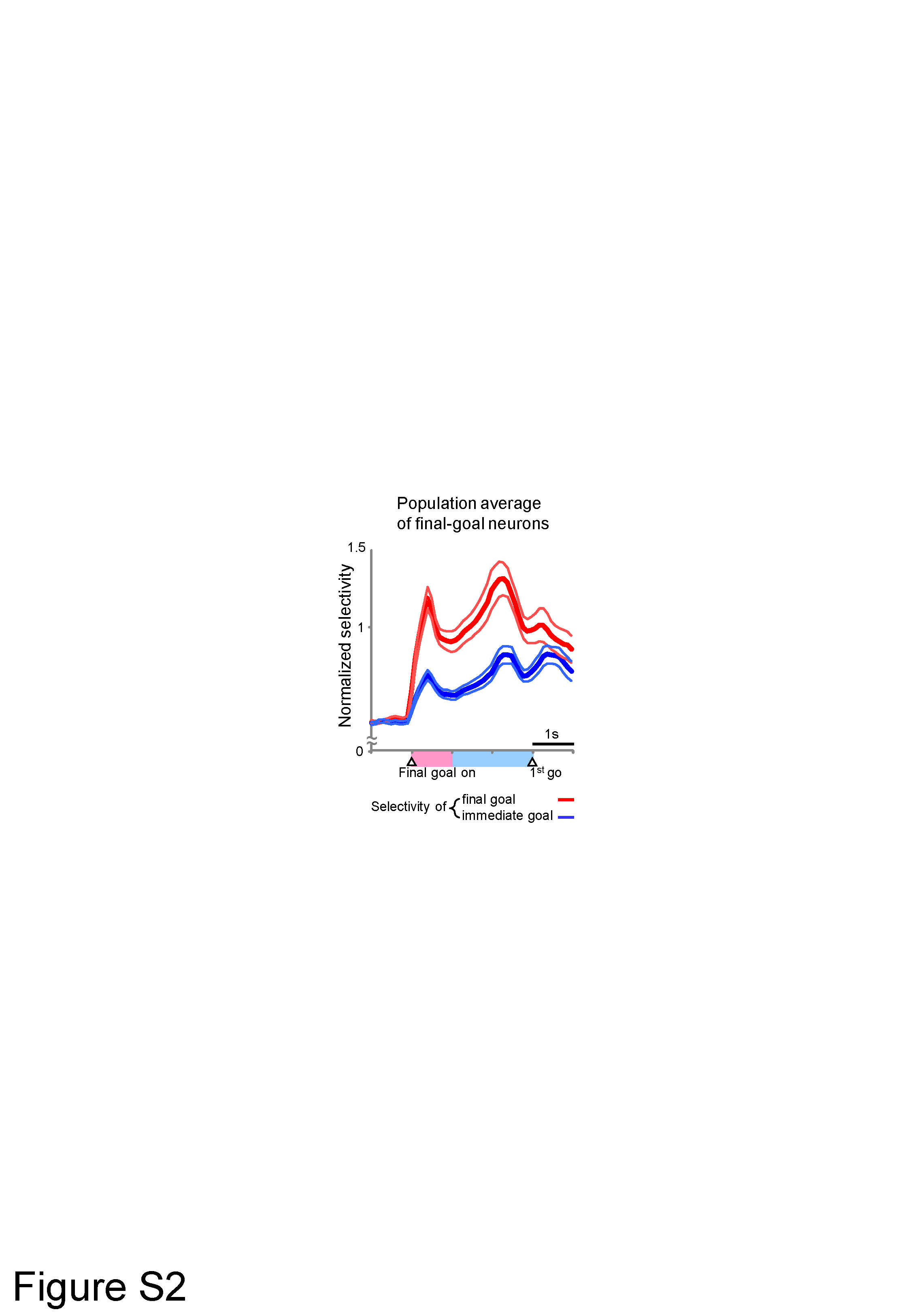

Supplement: Figure S2 — The lPFC neurons without showing representational transitions. The mean ± SEM selectivity for the final (red line) and immediate (blue) goals of the population of final-goal neurons (n = 259). The goal selectivity or regression coefficient is normalized to the significant level, P = 0.05. (TIF) [file pone.0080906.s002.tif]

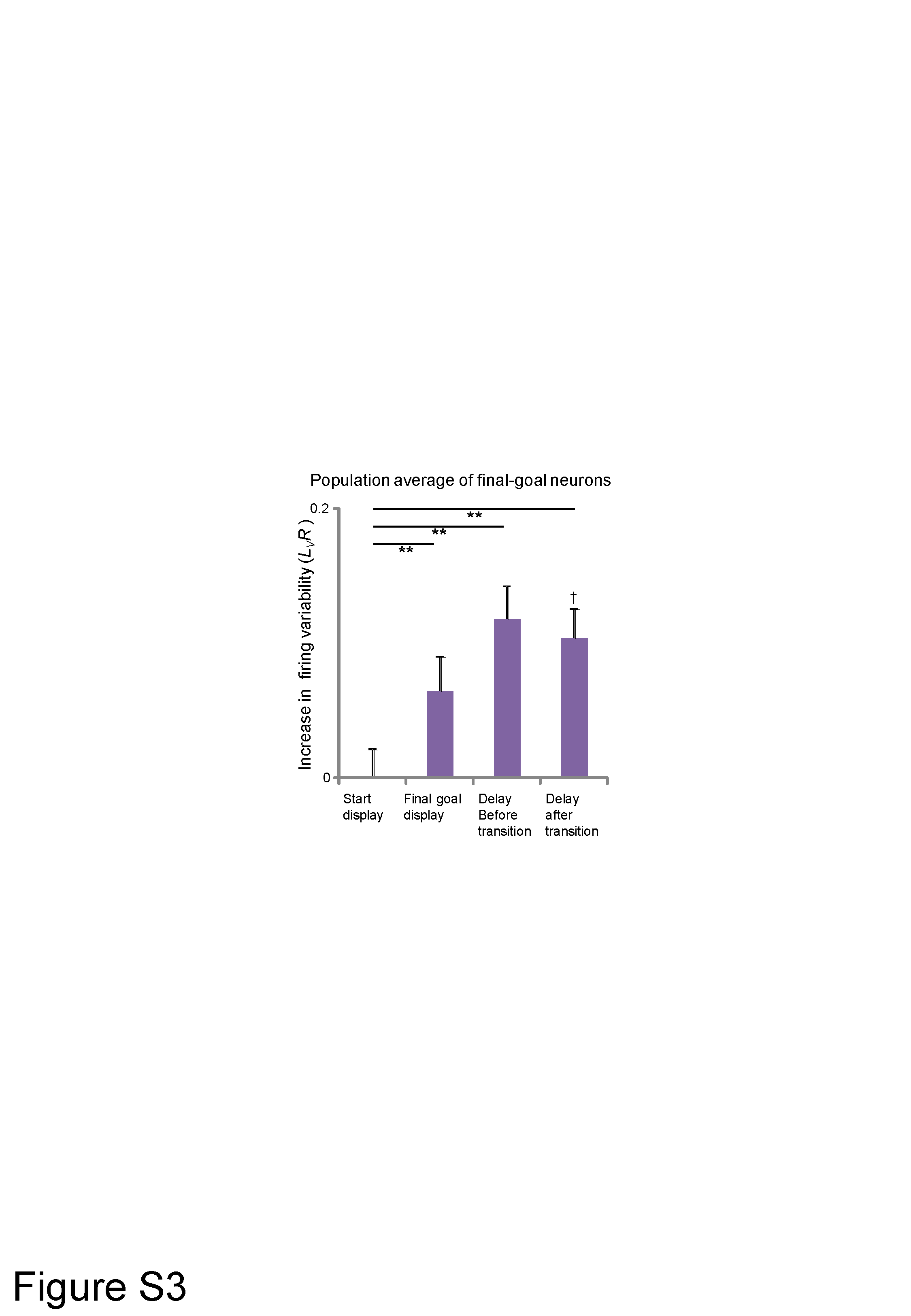

Supplement: Figure S3 — Firing variability changes in final-goal neurons. The average LVR increases in three epochs (final goal display, delay before transition, and delay after transition) from the initial value (1.17) in the start display is shown (n = 259). Start display, −700 to −800 ms; final goal display, 400 to 500 ms; delay before transitions, 1100 to 1200 ms from the final-goal onset; delay after transitions, 200 to 300 ms after the mean F-I transition time of F-I neurons. Error bars = SEM; *, P<0.05; **, P<0.01 (t-test) for comparisons between epochs. †, P<0.01 (t-test) for comparisons between final-goal and F-I neurons. (TIF) [file pone.0080906.s003.tif]

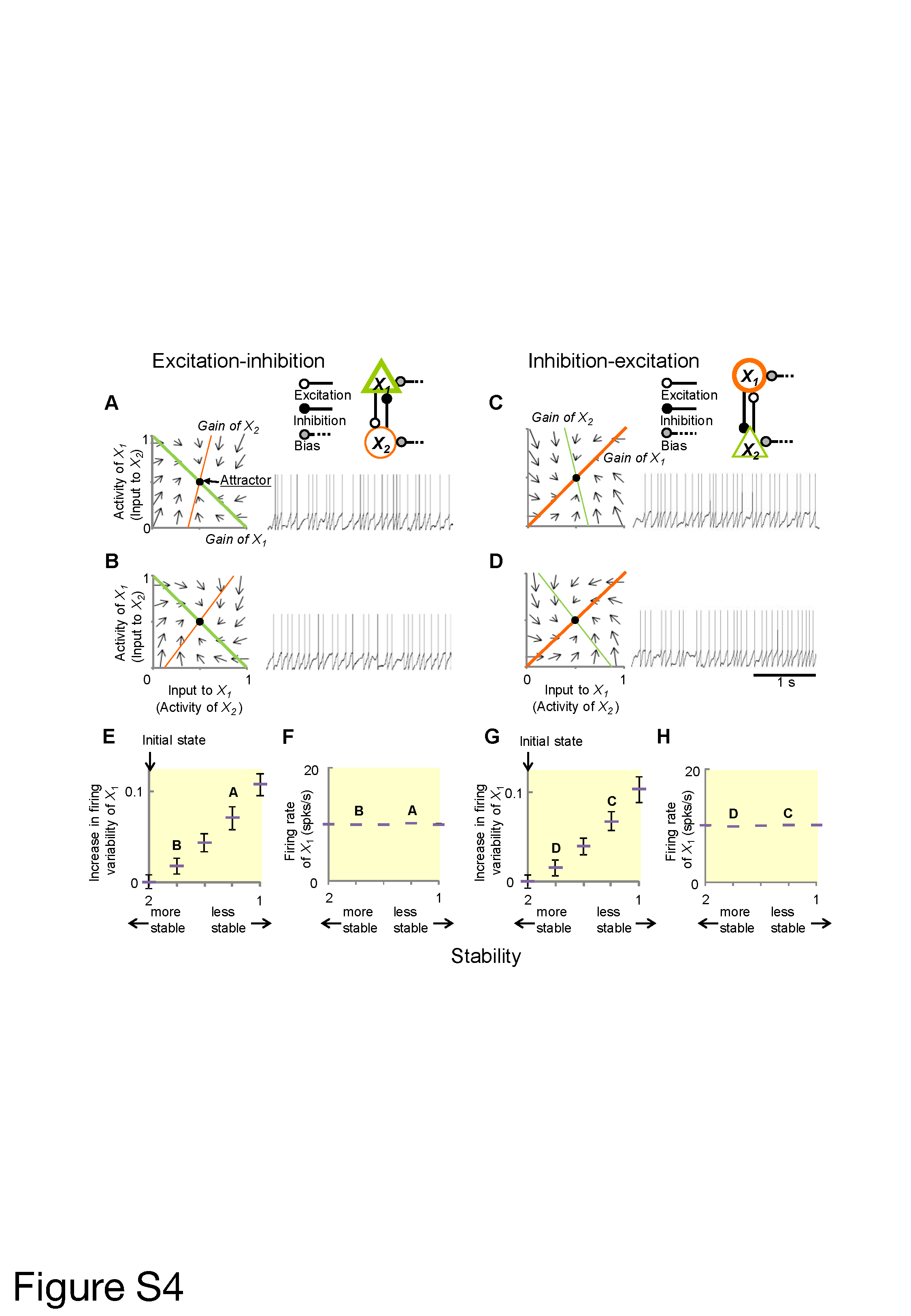

Supplement: Figure S4 — Changes in firing variability in excitation–inhibition networks. (A and B) Examples of phase-plane plots (left) of the nullclines for an excitation–inhibition network and firing patterns of a neuron associated with the network (right). Each node represents a neural population. The thick green line and thin orange line in the phase-plane plots are nullclines for nodes X 1 and X 2 respectively. The grey arrows indicate the vector fields. Examples of neuronal firing are in node X 1. Note that the gain functions of node X 1 in A and B are identical, whereas those of X 2 are changed. The value of 1 for the population activity corresponds to neuronal firing at 20 spikes/sec. (C and D), are the same figures for an inhibition–excitation network. The thick orange line and thin green line in the phase-plane plots are nullclines for nodes X 1 and X 2 respectively. (E–H) Systematic increases in firing variability from initial values (leftmost in E and G) with decreases in a stability measure “stiffness” (E and G) and without significant changes in firing rate (F and H). The firing variability of a neuron in X 2 exhibited similar results. (E and F), Excitation–inhibition; (G and H), Inhibition–excitation. Black circles in the phase–plane plots represent stable equilibrium points (point attractors). Error bars, SEM. (TIF) [file pone.0080906.s004.tif]

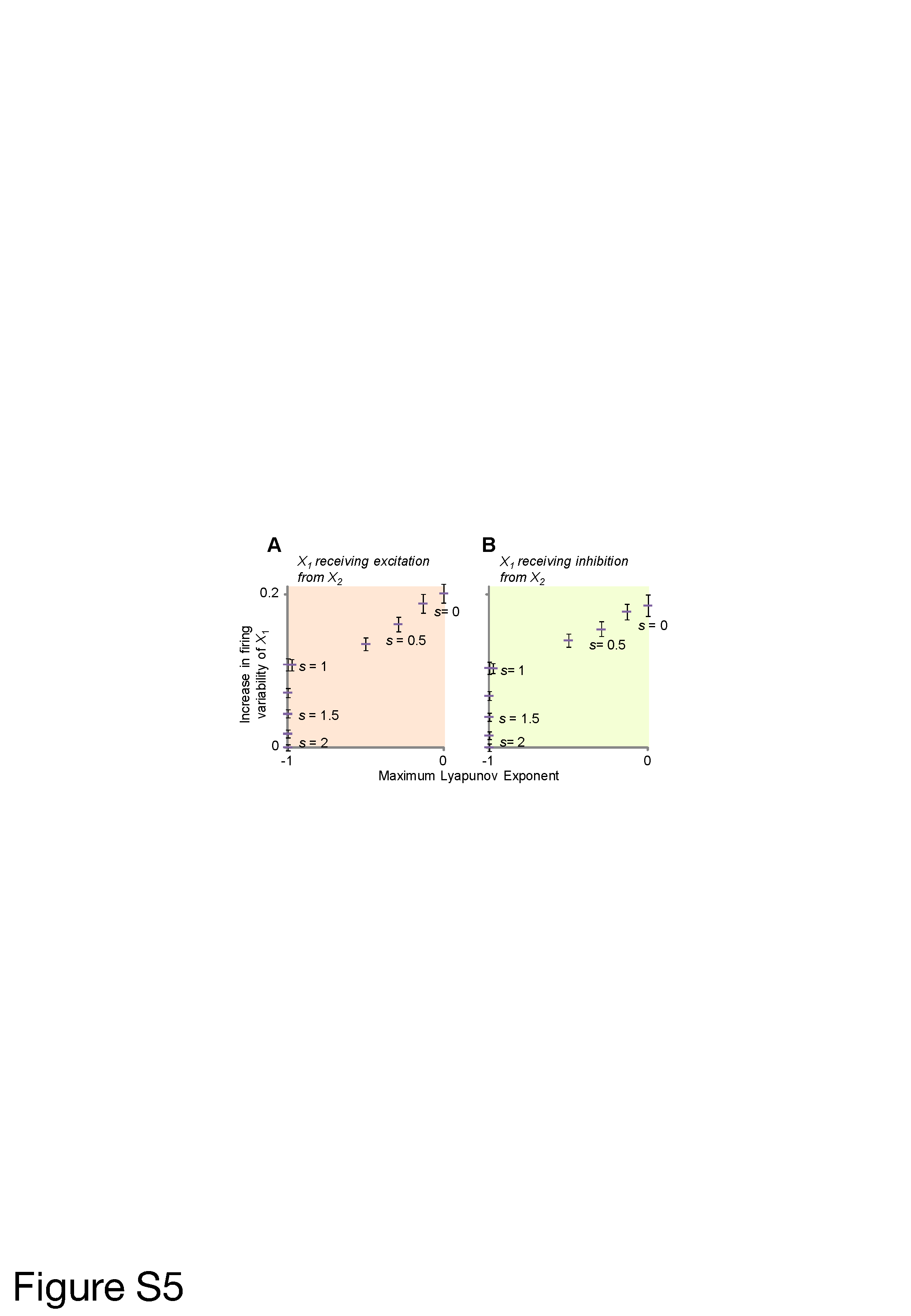

Supplement: Figure S5 — Consistency between stiffness and the maximum Lyapunov exponents. (A) Increases in LvR with stiffness, s, for cases where X1 received excitation from X2. Increases from the minimum value (s = 2.0) are plotted against the maximum Lyapunov exponent (MLE). Stiffness, s, was changed from 2.0 to 0.0 in 0.25 steps. The range of s from 2.0 to 21.0 corresponds to inhibition-excitation networks, and a re-plotting of the data in Fig. S4G. In this range of s, all MLEs were -1, because by definition they did not include the imaginary part of eigenvalues. In the range of s from 1.0 to 0.0 (where eigenvalues are not complex numbers, the networks are mutually excitatory, and the dynamics of networks do not include oscillatory components), stiffness and MLE have a one-to-one relationship. (B) As in (A) for cases where X1 received inhibition from X2. For the range of s from 2.0 to 1.0, the data in Fig. S4E were re-plotted (excitation-inhibition). Note that the range of s from 1.0 to 0.0 corresponds to mutual inhibitory networks. These data are consistent with (A). Error bars denote SEM. Parameters for these calculations can be found in the supplementary information. (TIF) [file pone.0080906.s005.tif]

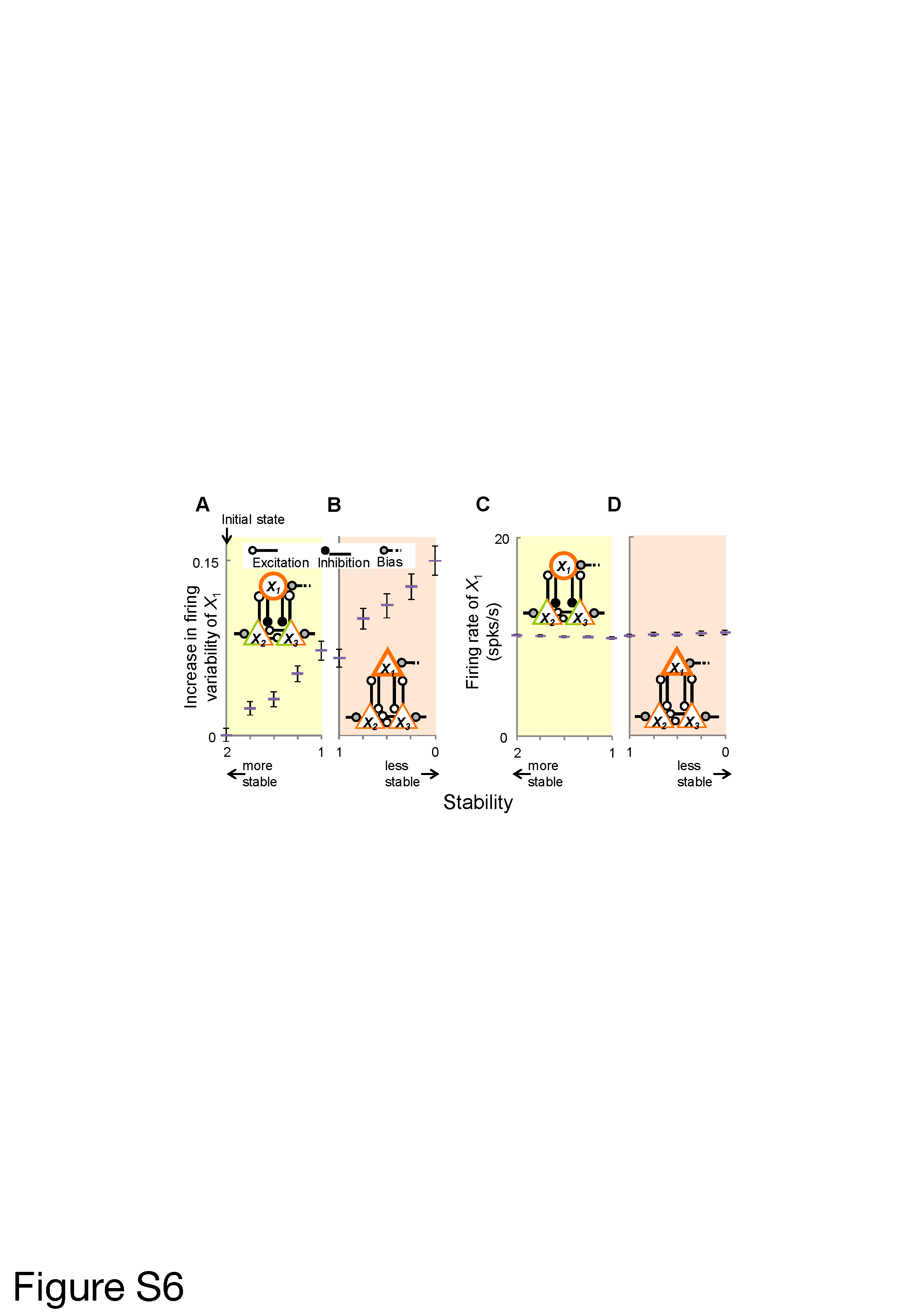

Supplement: Figure S6 — Changes in firing variability in three-node networks. The systematic increases in firing variability of a neuron in node X 1 from the initial value (leftmost in A) with decreases in the stability measure “stiffness” are plotted. (A) Inhibition–excitation–excitation. (B) Mutual excitation. Parameters of input functions were set for the network with a point attractor at (0.5, 0.5, 0.5), so that a neuron emitted spikes at approximately 10 spikes/sec. The gain function of X 1 was not changed in A and B, whereas those of X 2 and X 3 were changed and were identical. Note that these models exhibit systematic increases in firing variability as stability decreases (A and B) without significant changes in firing rate (C and D) across different network types, such as inhibition–excitation–excitation and mutual excitation. Error bars, SEM. (TIF) [file pone.0080906.s006.tif]

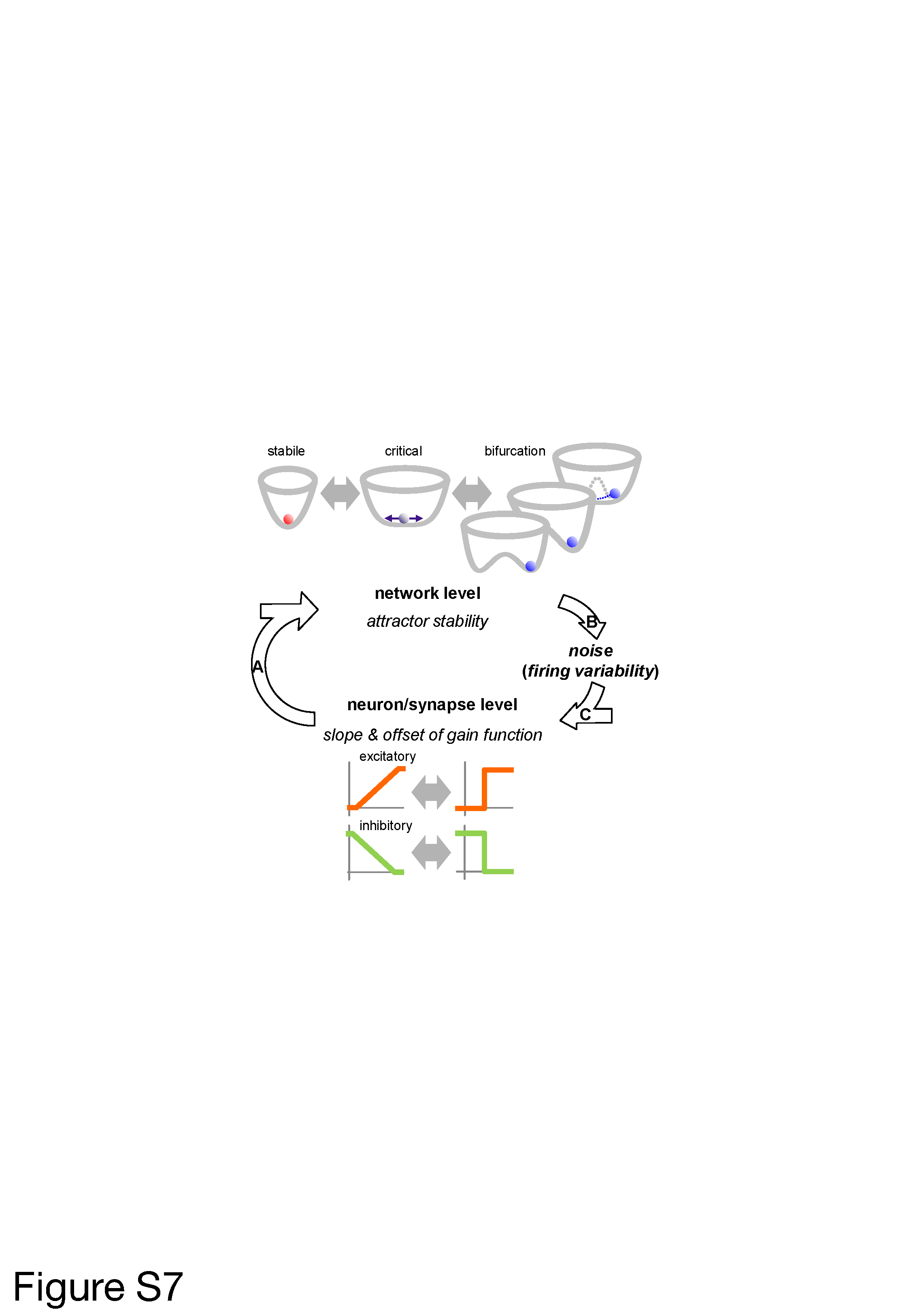

Supplement: Figure S7 — Proposed stability–gain interaction via noise. (A) Changes in neuronal gain functions determine the stability of the network and can cause bifurcations at the network level. (B) The state at the network level, particularly the stability of the attractor, can affect firing variability. (C) Firing variability can modulate the shape of the gain function determining the nullcline of the dynamics, in particular, its slope and offset, at the level of the neuron/synapse. (TIF) [file pone.0080906.s007.tif]
