# Supplementary material for: Increased Firing Irregularity as an Emergent Property of Neural-State Transition in Monkey Prefrontal Cortex
Source: PLoS One. 2013 Dec 4;8(12):e80906. doi: 10.1371/journal.pone.0080906 (PMC3857743; doi:10.1371/journal.pone.0080906)
Supplement: Text S1 — Parameters of model calculations. (DOC) [file pone.0080906.s008.doc]

**Supplementary Information**

**Parameters in each calculation**

**Figure 4**

Fig. 4E and F (mutual excitation):

Connectivity and noise:

*w_x_*_1_*_x_*_2_ = *w_x_*_2_*_x_*_1_ = 1.0, *w_x_*_1_*_x_*_1_ = *w_x_*_2_*_x_*_2_ = 0.0, and *σ* = 0.025.

Parameters of node *X*_1_:

*θ_x_*_1_ = 100.0, *c_x_*_1_ = 100.5, and *B_x_*_1_ = 0.0.

Parameters of node *X*_2_ (from l to r in Figs. 4E and F):

MLE -0.95: *θ_x_*_2_ =100.0, *c_x_*_2_ = 1.0, and *B_x_*_2_ = 100.0 (Fig. 4A, firing variability, 0.007);

MLE -0.75: *θ_x_*_2_ =100.0, *c_x_*_2_ = 7.25, and *B_x_*_2_ = 6.95;

MLE -0.5: *θ_x_*_2_ =100.0, *c_x_*_2_ = 25.5, and *B_x_*_2_ = 1.5;

MLE -0.25: *θ_x_*_2_ =100.0, *c_x_*_2_ = 57.5, and *B_x_*_2_ = 0.39 (Fig. 4B);

MLE -0.0: *θ_x_*_2_ =100.0, *c_x_*_2_ = 100.5, and *B_x_*_2_ = 0.0.

Fig. 4G and H (mutual inhibition):

Parameters of node *X*_1_:

*θ_x_*_1_ = 100.0, *c_x_*_1_ = 100.5, and *B_x_*_1_ = 1.0.

Parameters of node *X*_2_ were as follows (from l to r in Figs. 4G and H):

MLE -0.95: *θ_x_*_2_ = 100.0, *c_x_*_2_ = 1.0, and *B_x_*_2_ = 101.0(Fig. 4C, firing variability, 0.008);

MLE -0.75: *θ_x_*_2_ = 100.0, *c_x_*_2_ = 7.25, and *B_x_*_2_ = 7.95;

MLE -0.5: *θ_x_*_2_ = 100.0, *c_x_*_2_ = 25.5, and *B_x_*_2_ = 2.5;

MLE -0.25: *θ_x_*_2_ = 100.0, *c_x_*_2_ = 57.5, and *B_x_*_2_ = 1.39 (Fig. 4D);

MLE -0.0: *θ_x_*_2_ = 100.0, *c_x_*_2_ = 100.5, and *B_x_*_2_ = 1.0.

**Figure 5**

Fig. 5A (pitchfork bifurcation, mutual excitation):

Connectivity and noise:

*w_x_*_1_*_x_*_2_ = *w_x_*_2_*_x_*_1_ = 1.0, *w_x_*_1_*_x_*_1_ = *w_x_*_2_*_x_*_2_ = 0.0, and *σ* = 0.01.

Parameter of node *X_i_* (*i* = 1, 2):

Initial state: *θ_xi_* = 100.0, *c_xi_* = 50.5, and *B_xi_* = 0.5 (firing variability, 0.05);

Critical point: *θ_xi_* = 100.0, *c_xi_* = 100.5, and *B_xi_* = 0.0;

After transitions: *θ_xi_* = 100.0, *c_xi_* = 200.5, and *B_xi_* = -0.25.

Fig. 5B (pitchfork bifurcation, mutual inhibition):

Connectivity and noise:

*w_x_*_1_*_x_*_2_ = *w_x_*_2_*_x_*_1_ = -1.0, *w_x_*_1_*_x_*_1_ = *w_x_*_2_*_x_*_2_ = 0.0, and *σ* = 0.01.

Parameter of node *X_i_* (*i* = 1, 2):

Initial state: *θ_xi_* = 100.0; *c_xi_* = 50.5, and *B_xi_* = 1.5 (firing variability, 0.05);

Critical point: *θ_xi_* = 100.0, *c_xi_* = 100.5, and *B_xi_* = 1.0;

After transitions: *θ_xi_* = 100.0, *c_xi_* = 200.5, and *B_xi_* = 0.75.

**Figure 6**

Fig. 6A (saddle-node bifurcation, mutual excitation):

Connectivity and noise:

*w_x_*_1_*_x_*_2_ = *w_x_*_2_*_x_*_1_ = 1.0, *w_x_*_1_*_x_*_1_ = *w_x_*_2_*_x_*_2_ = 0.0, and *σ* = 0.01.

Parameter of node *X_i_* (*i* = 1, 2):

Initial state: *θ_xi_* = 100.0, *c_xi_* = 110.0, and *B_xi_* = 0.1 (firing variability, 0.01);

Critical point: *θ_xi_* = 100.0, *c_xi_* = 110.0, and *B_xi_* = -0.08;

After transitions: *θ_xi_* = 100.0, *c_xi_* = 110.0, and *B_xi_* = -0.2.

Fig. 6B (saddle-node bifurcation, mutual inhibition):

Connectivity and noise:

*w_x_*_1_*_x_*_2_ = *w_x_*_2_*_x_*_1_ = -1.0, *w_x_*_1_*_x_*_1_ = *w_x_*_2_*_x_*_2_ = 0.0, and *σ* = 0.01.

Parameter of node *X_i_* (*i* = 1, 2):

Initial state: *θ_xi_* = 100.0, *c_xi_* = 110.0, *B_x_*_1_ = 1.1, and *B_x_*_2_ = 0.9 (firing variability, 0.02);

Critical point: *θ_xi_* = 100.0, *c_xi_* = 110.0, *B_x_*_1_ = 1.1, and *B_x_*_2_ = 1.16;

After transitions: *θ_xi_* = 100.0, *c_xi_* = 110.0, *B_x_*_1_ = 1.1, and *B_x_*_2_ = 1.3.

Note that, in this case only, the lower limit of the gain function was set to 0.1 for the neuron to fire at a certain firing rate in order to examine the firing variability.

**Figure 7**

Fig. 7A (Hopf bifurcation, excitation–inhibition):

Connectivity and noise:

Initial state: *w_x_*_1_*_x_*_2_ = -1.3, *w_x_*_2_*_x_*_1_ = 1.3, and *w_x_*_1_*_x_*_1_ = *w_x_*_2_*_x_*_2_ = 0.0 and *σ* = 0.01;

Critical point: *w_x_*_1_*_x_*_2_ = -1.3, *w_x_*_2_*_x_*_1_ = 1.3, *w_x_*_1_*_x_*_1_ = 2.0, and *w_x_*_2_*_x_*_2_ = 0.0 and *σ* = 0.01;

After transitions: *w_x_*_1_*_x_*_2_ = -1.3, *w_x_*_2_*_x_*_1_ = 1.3, *w_x_*_1_*_x_*_1_ = 2.1, and *w_x_*_2_*_x_*_2_ = 0.0 and *σ* = 0.01.

Parameter of node *X_i_* (*i* = 1, 2):

Initial state: *θ_xi_* = 100.0, *c_xi_* = 100.0, *B_x_*_1_ = 1.15, *B_x_*_2_ = -0.15 (firing variability, 0.02);

Critical point: *θ_xi_* = 100.0, *c_xi_* = 100.0, *B_x_*_1_ = 0.15, *B_x_*_2_ = -0.15;

After transitions: *θ_xi_* = 100.0, *c_xi_* = 100.0, *B_x_*_1_ = 0.1, *B_x_*_2_ = -0.15.

Fig.7B (Hopf bifurcation, inhibition–excitation):

All parameters were the reverse of the case Fig.7A. Firing variability at initial state was 0.02.

**Figure S4**

Fig. S4E and F (excitation–inhibition):

Connectivity:

*w_x_*_1_*_x_*_2_ = -1.0, *w_x_*_2_*_x_*_1_ = 1.0, and *w_x_*_1_*_x_*_1_ = *w_x_*_2_*_x_*_2_ = 0.0.

Parameters of node *X*_1_ (fixed) and noise:

*θ_x_*_1_ = 1000.0, *c_x_*_1_ = 1000.5, *B_x_*_1_ = 1.0, and *σ* = 0.025.

Parameters of node *X*_2_ (from l to r):

stiffness 2.0: *θ_x_*_2_ =1000.0, *c_x_*_2_ = 1000.5, and *B_x_*_2_ = 0.0 (firing variability, 0.15);

stiffness 1.75: *θ_x_*_2_ =1000.0, *c_x_*_2_ = 750.5, and *B_x_*_2_ = 0.17 (same as Fig. S4B);

stiffness 1.5: *θ_x_*_2_ =1000.0, *c_x_*_2_ = 500.5, and *B_x_*_2_ = 0.5;

stiffness 1.25: *θ_x_*_2_ =1000.0, *c_x_*_2_ = 250.5, and *B_x_*_2_ = 1.5 (same as Fig. S4A);

stiffness 1.0: *θ_x_*_2_ =1000.0, *c_x_*_2_ = 1.5, and *B_x_*_2_ = 499.5.

Fig.S4G and H (inhibition–excitation):

Connectivity:

*w_x_*_1_*_x_*_2_ = 1.0, *w_x_*_2_*_x_*_1_ = -1.0, and *w_x_*_1_*_x_*_1_ = *w_x_*_2_*_x_*_2_ = 0.0.

Parameters of node *X*_1_ (fixed) and noise:

*θ_x_*_1_ = 1000.0, *c_x_*_1_ = 1000.5, *B_x_*_1_ = 0.0, and *σ* = 0.025.

Parameters of node *X*_2_ (from l to r):

stiffness 2.0: *θ_x_*_2_ =1000.0, *c_x_*_2_ = 1000.5, and *B_x_*_2_ = 1.0 (firing variability, 0.015);

stiffness 1.75: *θ_x_*_2_ =1000.0, *c_x_*_2_ = 750.5, and *B_x_*_2_ = 1.17 (same as Fig. S4D);

stiffness 1.5: *θ_x_*_2_ =1000.0, *c_x_*_2_ = 500.5, and *B_x_*_2_ = 1.5;

stiffness 1.25: *θ_x_*_2_ =1000.0, *c_x_*_2_ = 250.5, and *B_x_*_2_ = 2.5 (same as Fig. S4C);

stiffness 1.0: *θ_x_*_2_ =1000.0, *c_x_*_2_ = 1.5, and *B_x_*_2_ = 500.5.

**Figure S5**

Fig. S5A

For the range of *s* from 2.0 to 1.0 (inhibition-excitation networks), the parameters were identical to those in Fig. S4G. For the range of *s* from 1.0 to 0.0 (mutual excitation networks), the parameters were as follows:

Connectivity:

*w_x_*_1_*_x_*_2_ = 1.0, *w_x_*_2_*_x_*_1_ = 1.0, and *w_x_*_1_*_x_*_1_ = *w_x_*_2_*_x_*_2_ = 0.0

Parameters of node *X*_1_ (fixed) and noise:

*θ_x_*_1_ = 1000.0, *c_x_*_1_ = 1000.5, *B_x_*_1_ = 0.0, and *σ* = 0.025.

Parameters of node *X*_2_ (from l to r):

stiffness 1.0: *θ_x_*_2_ =1000.0, *c_x_*_2_ = 1.5, and *B_x_*_2_ = 499.5;

stiffness 0.75: *θ_x_*_2_ =1000.0, *c_x_*_2_ = 250.5, and *B_x_*_2_ = 1.5;

stiffness 0.5: *θ_x_*_2_ =1000.0, *c_x_*_2_ = 500.5, and *B_x_*_2_ = 0.5;

stiffness 0.25: *θ_x_*_2_ =1000.0, *c_x_*_2_ = 750.5, and *B_x_*_2_ = 0.17;

stiffness 0.0: *θ_x_*_2_ =1000.0, *c_x_*_2_ = 1000.5, and *B_x_*_2_ = 0.0.

Fig. S5B

For the range of *s* from 2.0 to 1.0 (excitation-inhibition networks), the parameters were identical to those in Fig. S4E. For the range of *s* from 1.0 to 0.0 (mutual inhibition networks), the parameters were as follows:

Connectivity:

*w_x_*_1_*_x_*_2_ = -1.0, *w_x_*_2_*_x_*_1_ = -1.0, and *w_x_*_1_*_x_*_1_ = *w_x_*_2_*_x_*_2_ = 0.0.

Parameters of node *X*_1_ (fixed) and noise:

*θ_x_*_1_ = 1000.0, *c_x_*_1_ = 1000.5, *B_x_*_1_ = 1.0, and *σ* = 0.025.

Parameters of node *X*_2_ (from l to r):

stiffness 1.0: *θ_x_*_2_ =1000.0, *c_x_*_2_ = 1.5, and *B_x_*_2_ = 500.5;

stiffness*θ_x_*_2_ =1000.0, *c_x_*_2_ = 250.5, and *B_x_*_2_ = 2.5;

stiffness 0.5: *θ_x_*_2_ =1000.0, *c_x_*_2_ = 500.5, and *B_x_*_2_ = 1.5;

stiffness 0.25: 0.75: *θ_x_*_2_ =1000.0, *c_x_*_2_ = 750.5, and *B_x_*_2_ = 1.17;

stiffness 0.0:*θ_x_*_2_ =1000.0, *c_x_*_2_ = 1000.5, and *B_x_*_2_ = 1.0.

**Figure S6**

Parameters of node *X*_1_ (fixed in A and B) and noise:

*θ_x_*_1_ = 1000.0, *c_x_*_1_ = 1000.5, *B_x_*_1_ = -0.5, and *σ* = 0.025.

Fig. S6A and C:

Connectivity:

*w_x_*_2_*_x_*_1_ = *w_x_*_3_*_x_*_1_ =-1, *w_x_*_1_*_x_*_2_ = *w_x_*_1_*_x_*_3_ = *w_x_*_2_*_x_*_3_ = *w_x_*_3_*_x_*_2_= 1.0, and *w_x_*_1_*_x_*_1_ = *w_x_*_2_*_x_*_2_ = *w_x_*_3_*_x_*_3_ = 0.0.

Parameters of node *X*_2_ and *X*_3_ (from l to r):

stiffness 2.0: *θ_x_*_2or3_ =1000.0, *c_x_*_2or3_ = 416.0, and *B_x_*_2or3_ = 1.20 (firing variability, 0.21);

stiffness 1.75: *θ_x_*_2or3_ =1000.0, *c_x_*_2or3_ = 324.2, and *B_x_*_2or3_ = 1.54;

stiffness 1.5: *θ_x_*_2or3_ =1000.0, *c_x_*_2or3_ = 226.0, and *B_x_*_2or3_ = 2.22;

stiffness 1.25: *θ_x_*_2or3_ =1000.0, *c_x_*_2or3_ = 119.5, and *B_x_*_2or3_ = 4.20;

stiffness 1.0: *θ_x_*_2or3_ =1000.0, *c_x_*_2or3_ = 1.0, and *B_x_*_2or3_ = 1000.0.

Fig. S6B and D:

Connectivity:

*w_x_*_1_*_x_*_2_ = *w_x_*_1_*_x_*_3_ = *w_x_*_2_*_x_*_1_= *w_x_*_2_*_x_*_3_ = *w_x_*_3_*_x_*_1_ = *w_x_*_3_*_x_*_2_ = 1.0, and *w_x_*_1_*_x_*_1_ = *w_x_*_2_*_x_*_2_ = *w_x_*_3_*_x_*_3_ = 0.0.

Parameters of node *X*_2_ and *X*_3_ (from l to r):

stiffness 1.0: *θ_x_*_2or3_ =1000.0, *c_x_*_2or3_ = 1.0, and *B_x_*_2or3_ = 999.0;

stiffness 0.75: *θ_x_*_2or3_ =1000.0, *c_x_*_2or3_ = 108.5, and *B_x_*_2or3_ = 3.63;

stiffness 0.5: *θ_x_*_2or3_ =1000.0, *c_x_*_2or3_ = 194.5, and *B_x_*_2or3_ = 1.58;

stiffness 0.25: *θ_x_*_2or3_ =1000.0, *c_x_*_2or3_ = 268.5, and *B_x_*_2or3_ = 0.87;

stiffness 0.0: *θ_x_*_2or3_ =1000.0, *c_x_*_2or3_ = 333.0, and *B_x_*_2or3_ = 0.50.
